# Virtual Reality Simulation in Postgraduate Pediatric Critical Care Training Based on Trainee Perceptions in London: Exploratory Mixed Methods Study

**DOI:** 10.2196/85743

**Published:** 2026-06-25

**Authors:** Anne Claudia Berlin, David Harrison, Rima Chakrabarti

**Affiliations:** 1 LMU Munich, Munich, Germany; 2 University College Medical School, London, United Kingdom; 3 Royal College of Physicians, St Andrews Place, London, NW14LE, United Kingdom; 4 Barnet Hospital Trust, University College London, Gower Street, London, WC1E 6BT, United Kingdom, 44 20 3108 8235

**Keywords:** medical education, simulation, virtual reality, clinical training, pediatric critical care, pediatrics

## Abstract

**Background:**

Simulation-based training has established itself as integral to clinical education, particularly for high-stakes, low-frequency pediatric emergencies. Innovations incorporating virtual reality (VR) are rapidly gaining traction for offering scalable, repeatable, and immersive opportunities for scenario-based learning. Understanding its role and applicability in postgraduate pediatric training, however, remains limited, with further exploration required into how pediatric trainees perceive, conceptualize, and anticipate VR-based simulation within real-world training contexts.

**Objective:**

This study explored London-based pediatric trainees’ perceptions of VR simulation as an adjunct for developing skills in recognizing and managing critically ill children.

**Methods:**

An exploratory mixed methods study was conducted among pediatric trainees across all training levels within the London School of Paediatrics between April 2024 and July 2024. Data were collected using a 35-item online questionnaire containing Likert-scale, categorical, and open-ended questions, alongside virtual semistructured interviews. The questionnaire explored current training practices; confidence and preparedness in managing critically ill children; familiarity and experience with VR; and perceived benefits, limitations, barriers, and facilitators to adoption. Quantitative data were analyzed descriptively, with exploratory Mann-Whitney *U* tests and Spearman correlations where appropriate. Internal consistency of key domains was assessed using Cronbach α. Qualitative data from open-ended responses and interviews were analyzed thematically using the Braun and Clarke reflexive approach. Quantitative and qualitative strands were integrated at the interpretation stage to contextualize survey patterns with illustrative qualitative insights.

**Results:**

Thirty trainees participated in the survey (30/450, 6.7%; female: 16/30, 53%), with participants spanning all 8 training years. Two senior trainees participated in interviews. Clinical exposure or experience and simulation training were identified as central to developing skills in managing pediatric emergencies. Trainees also described limited exposure to high-acuity scenarios; variable access to high-fidelity simulation; and constraints related to workload, supervision, and feedback. Most participants (21/30) had no prior VR exposure in a medical setting, while 17% (5/30) had used VR training, and all reported positive experiences. Despite limited exposure, 93% (28/30) of participants were willing to try VR simulation for exposure to rare scenarios, structured decision-making, and confidence-building. Key perceived barriers included high cost (24/30, 80%), technological literacy (17/30, 57%), infrastructure (15/30, 50%), and limited stakeholder familiarity or support (25/30, 83%). Participants suggested taster sessions, faculty advocacy, leadership engagement, and phased implementation as potential facilitators. Internal consistency of attitudinal survey items was good (Cronbach α=0.80).

**Conclusions:**

Despite limited exposure, pediatric trainees viewed VR simulation as a valuable adjunct to existing critical care training, particularly for those in earlier stages of training. However, these findings represent anticipatory perceptions rather than evidence of educational effectiveness. The implementation of VR will depend on addressing key infrastructural, organizational, educational, and equity-related barriers. Further multicenter studies are needed to evaluate the educational impact and feasibility, learning outcomes, and cost-effectiveness in postgraduate pediatric critical care training programs.

## Introduction

Managing critically ill patients is a vital yet complex skill, particularly in pediatrics, where prompt, multidisciplinary intervention is required to reduce high morbidity and mortality [1]. Competency in these high-stakes, low-frequency scenarios relies on the integration of theoretical knowledge, clinical reasoning, decision-making, and psychomotor skills [2]. Medical education aims to develop these competencies through a combination of theoretical teaching, clinical experience, and simulation-based education [1,3,4].

Simulation training, particularly with high-fidelity physical simulators, is widely regarded as a key modality for supporting the development of emergency skills in pediatric education, offering immersive environments for practicing clinical and decision-making skills [1,5]. Although simulation-based training using high-fidelity simulators has been available and systematically studied for more than 20 years, its implementation in routine pediatric education remains limited [6,7]. Barriers such as cost, faculty availability, the need for experienced trainers, access limitations, and extended working hours continue to hinder wider use [5,8]. In response, virtual reality (VR) technology has recently emerged as a potentially scalable and cost-effective adjunct to traditional high-fidelity simulation, aiming to mitigate some of these limitations in medical training [9-11].

VR may enable learners to engage in immersive, interactive clinical scenarios and support the rehearsal of practical, procedural, and cognitive skills under simulated stress within a controlled environment [10-12]. Its adaptability offers a range of repeatable, customizable scenarios, potentially supporting self-directed learning, repeated practice, and anticipated skill development [7,11]. Trainees can perform patient examinations, history-taking, and interventions with simulated feedback and structured debriefing, although the extent and quality of such feedback vary across platforms [10,11].

In the United Kingdom, more than 50 Health Education England organizations, including the East of England deanery, have integrated VR simulation into undergraduate, health professional, and foundation training programs, often using platforms such as Oxford Medical Simulation [13].

Despite its increasing uptake, studies exploring the role of VR-based simulation in postgraduate pediatric emergency and critical care training remain limited. While research on VR-based technology has predominantly focused on feasibility and accessibility, the economic and educational outcomes, as well as how VR is perceived, conceptualized, and integrated within postgraduate training contexts, have been recognized yet remain underexplored [14,15].

Understanding trainees’ perceptions is particularly important in this setting. Pediatric trainees, however, often have limited direct exposure to VR-based simulation, and their expectations may influence both engagement and the successful implementation of such technologies [16]. Exploring these anticipatory perceptions may, therefore, provide valuable insights into the potential role of VR as an adjunct to existing training approaches, as well as inform the design of future educational interventions.

Drawing on insights from trainees within the London School of Paediatrics (LSP; London deanery), this study aims to explore how VR-based simulation is conceptualized and anticipated, and—where applicable—experienced as a supplementary tool for pediatric critical care training, with particular attention to perceived preparedness, confidence, and training needs. By situating these perspectives within a real-world postgraduate training program, the study provides context-specific insights into the perceived value, acceptability, and potential integration of VR-based simulation, thereby informing future curriculum design and evaluation strategies.

## Methods

### Study Design and Research Approach

This exploratory sequential mixed methods study examined pediatric trainees’ perceptions of VR simulation as a supplementary tool for developing skills relevant to managing critically ill children. The study was informed by a broadly constructivist paradigm, recognizing that trainees’ perceptions are shaped by individual experiences, learning environments, and contextual constraints [17]. Elements of pragmatism guided methodological choices, reflecting the study’s emphasis on generating practically useful insights to inform real-world training design [17].

A mixed methods approach was selected to capture both the breadth of trainees’ perceptions through quantitative survey data and the depth of their underlying reasoning through qualitative interviews [18]. Quantitative and qualitative components were collected iteratively, allowing early insights to shape later data collection. This alignment between the paradigm and methodology supported a holistic examination of trainees’ conceptualizations of VR-based learning.

Quantitative and qualitative data were integrated primarily at the interpretation stage. Survey findings provided an overview of the distribution of trainees’ perceptions, levels of VR familiarity, perceived educational value, and barriers to implementation. Qualitative data from open-ended survey responses and interviews were then used to contextualize and explain these patterns, particularly where survey responses suggested uncertainty, divergence, or limited prior experience. This approach enabled triangulation between numerical response patterns and participants’ narrative accounts, while recognizing that the qualitative component was exploratory and illustrative rather than intended to achieve saturation or generate an independent theory.

### Setting, Participants, and Recruitment

Data were collected between April 2024 and July 2024 within the LSP. The LSP is a large and heterogeneous postgraduate deanery comprising approximately 450 trainees (specialty trainees [STs]: ST1-ST8, corresponding to UK postgraduate 1-8 y pediatric specialty training) across a wide range of pediatric subspecialties. Inclusion across all training levels was intended to capture perspectives shaped by differing exposure to high-acuity clinical environments and simulation-based education.

A nonprobability, convenience sampling strategy was used, which was appropriate for an exploratory, student-led project focused on eliciting individual experiences. Recruitment occurred via the LSP’s official WhatsApp groups, where the participant information sheet, survey link, and optional interview registration link were distributed (see Multimedia Appendix 1 for the recruitment message). Invitations were issued periodically during the data collection period to mitigate the low participation rates common among postgraduate trainees.

As the lead researcher was based outside the United Kingdom, all data were collected online. This format enabled participation across geographically dispersed hospital sites, reduced scheduling barriers for busy clinicians, and preserved anonymity for survey responses. Completion of the questionnaire constituted implied consent, while interview participants provided written informed consent. The researcher had no prior professional relationship with the participants, minimizing perceived coercion.

### Survey Instrument and Domains

A 35-item online questionnaire was developed to assess five domains relevant to postgraduate pediatric training and VR adoption: (1) participant characteristics and training background, (2) current approaches to skill development and managing critically ill children, (3) familiarity with artificial intelligence (AI) and VR, (4) perceived educational value of VR simulation in pediatric critical care training, and (5) perceived barriers and facilitators to its adoption and implementation. These domains informed the structure of the results analysis. A selection of open-ended questions was included in the questionnaire to capture qualitative elaboration. These questions remained optional, while the remaining questions required completion prior to submission.

### Outcomes and Measures

Primary outcomes included trainees’ perceptions of the educational value of VR simulation, including their perceived usefulness, realism, and willingness to engage with VR-based training.

Secondary outcomes included perceived preparedness and confidence in managing critically ill children with the current approaches to skill development, familiarity with AI and VR technologies, and perceived barriers to and facilitators of VR implementation.

Items were primarily assessed using Likert-type scales, supplemented with categorical and open-ended responses.

### Instrument Development and Theoretical Foundation

A 35-item online questionnaire (Microsoft Forms) was developed, following a focused literature review and consultation with UK pediatric trainees, simulation educators, and a pediatric critical care lead [19].

Item development was theory-informed. The technology acceptance model shaped items addressing perceived usefulness, ease of use, and intention to adopt VR [20]. Diffusion of innovations theory guided items related to relative advantage, compatibility, and barriers to implementation. Experiential learning theory [21] and self-efficacy theory [22] informed items related to preparedness, confidence, and perceived learning. Simulation fidelity and immersion frameworks informed items assessing anticipated realism and presence. This approach was adopted because it was anticipated that some participants would have limited prior exposure to VR. A brief descriptive paragraph introduced VR simulation, but no extensive explanatory materials were provided to avoid unduly biasing participants’ responses.

### Expert Review and Piloting

The questionnaire and interview guide underwent expert review by a medical education specialist (RC) and an independent pediatric critical care consultant to assess face and content validity. The reviewers evaluated item clarity, theoretical alignment, conceptual grouping, and coverage of key domains. Feedback informed revisions to item wording, ordering, and the balance of open-ended and closed-ended items. Subsequent piloting with 3 pediatric trainees focused on clarity, cognitive load, usability, and estimated completion time. Minor refinements were made to improve flow and accessibility. Pilot participants were excluded from the main study. The full questionnaire is provided in Multimedia Appendix 2, and the participant information sheet and consent form are provided in Multimedia Appendix 3.

### Psychometric Considerations

As the instrument was exploratory and not intended as a validated psychometric scale, psychometric evaluation focused on internal consistency and item performance rather than formal scale validation. The Cronbach α was calculated for the attitudinal item set as a whole, yielding a value of approximately 0.80, which suggests good internal consistency for an exploratory educational instrument. Subscale α values were not calculated due to sample size constraints (n=30) and because the questionnaire was not designed for formal scale development. Item distribution patterns, including skewness, ceiling and floor effects, and corrected item-total correlations, were examined to contextualize interpretation.

### Qualitative Component: Interview Procedures

Semistructured interviews were selected to explore trainees’ perspectives in greater depth while maintaining thematic coherence. Focus groups were initially planned; however, limited volunteer availability prompted an ethics-approved amendment to conduct individual interviews. Interviews were conducted via Microsoft Teams, audiorecorded with consent, and transcribed verbatim. The interview guide was developed from the questionnaire domains to ensure conceptual alignment (Multimedia Appendix 4). Qualitative components were intended to be illustrative and exploratory, providing contextual depth and illustrative insight rather than confirmatory evidence.

### Data Analysis

Only fully submitted questionnaires were included in the analysis; missing data within optional open-ended items were treated as item-level nonresponse.

#### Quantitative Analysis

Descriptive statistics (frequencies, percentages, medians, and IQRs) were generated using SPSS (version 29; IBM) and Microsoft Excel (version 16.66.1; Microsoft). Given the ordinal nature of Likert-scale responses, questionnaire-derived quantitative data were summarized using medians and IQRs.

Item-level response distributions, including skewness and ceiling or floor effects, were examined. Internal consistency was estimated using Cronbach α. Exploratory inferential analyses appropriate for the sample size and data structure included Mann-Whitney *U* tests for subgroup comparisons and Spearman rank-order correlations to explore descriptive associations. These analyses were interpreted descriptively and as hypothesis-generating.

#### Qualitative Analysis

Qualitative data from open-ended survey responses and interview transcripts were analyzed using the reflexive thematic analysis approach developed by Braun and Clarke [23,24]. The analysis followed an iterative 6-phase process involving familiarization with the data, generation of initial codes, development and review of themes, refinement and naming of themes, and selection of illustrative quotations.

Given the theory-informed design of the questionnaire and interview guide, a predominantly deductive coding strategy was used. Initial coding was informed by the study’s predefined domains and theoretical foundations, including perceived usefulness, preparedness, confidence, barriers, and facilitators to VR adoption. At the same time, the analysis remained open to inductive insights and divergent perspectives that extended or nuanced these domains. Theme development, therefore, represented an interpretive process of organizing and contextualizing participants’ perspectives rather than the identification of entirely emergent themes.

Consistent with the Braun and Clarke reflexive approach, themes were understood as researcher-generated interpretive outputs developed through engagement with the data and informed by the study’s theoretical framework. Qualitative findings were primarily intended to provide contextual depth and explanatory insight for survey findings rather than to generate a comprehensive, stand-alone theoretical account.

Reflexivity was supported through analytic memorandum writing, documentation of coding decisions, and ongoing consideration of the lead researcher’s positionality as a pediatric trainee and medical education researcher with an interest in simulation-based learning. This positionality may have sensitized the researcher to the perceived educational value of simulation and VR-based education, while requiring conscious attention to alternative, more skeptical perspectives.

To enhance credibility, interpretations were iteratively reviewed against both survey free-text responses and interview transcripts. Dependability was supported through the maintenance of a documented decision trail that outlined coding refinements and theme development. Confirmability was strengthened through transparent linkage between interpretations and supporting participant quotations. Transferability was addressed by providing detailed contextual information on the LSP, participant characteristics, and the current stage of VR adoption in postgraduate pediatric training, enabling readers to assess the relevance of the findings to other educational settings.

### Ethical Considerations

This study was conducted as part of an MSc in Medical Education at University College London. Ethical approval was granted by University College London’s Ethics Committee (reference number: 27235.001) via a low-risk application. Participation was voluntary, with all data anonymized using ID codes and securely managed, with minimal personal data collected. Completion of the online questionnaire constituted implied consent, while interview participants provided written informed consent before participation. No financial or nonfinancial compensation was provided to participants. Reporting of the online survey followed the CHERRIES (Checklist for Reporting Results of Internet E-Surveys) guideline (Checklist 1) [25].

All procedures adhered to the General Data Protection Regulation and British Educational Research Association guidelines. The training program director of the LSP approved trainee recruitment. Informed consent was obtained, and interviewees were given the option to withdraw within 4 weeks.

## Results

### Overview

Survey (participation rate: 30/450, 6.7%) and interview (n=2) data generated five interrelated themes: (1) participant characteristics and current training, (2) skill development approaches and their limitations, (3) familiarity with AI and VR, (4) anticipated educational value of VR simulation for managing critically ill children, and (5) perceived barriers and facilitators to implementation. Most trainees had limited or no prior VR simulation experience, and their responses, therefore, reflect expectations about VR’s potential value rather than evaluations of actual VR-based training. Given the low participation rate and small VR-experienced subgroup, findings should be interpreted cautiously as exploratory and indicative only, not representative of the wider trainee population.

### Psychometric Properties of the Survey

Internal consistency of the attitudinal item set was good (Cronbach α=0.80), suggesting acceptable reliability for this exploratory educational instrument. Corrected item-total correlations were generally satisfactory, with most items exceeding the recommended threshold of 0.30. A small number of items demonstrated lower or negative item-total correlations, likely reflecting conceptual heterogeneity and response clustering commonly observed in perception-focused educational surveys. Ceiling effects were evident for several items assessing perceived educational usefulness and realism, while some barrier-related items showed floor-pattern clustering. These response patterns were considered when interpreting attitudinal findings, and detailed psychometric analyses are provided in Multimedia Appendix 5.

### Participant Characteristics

Of approximately 450 eligible trainees within the LSP deanery, 30 completed the survey (participation rate: 6.7%), with 16 out of 30 (53%) identified as female. Respondents spanned all training levels (ST1-ST8) and represented a broad range of pediatric subspecialty aspirations. Detailed participant characteristics are provided in Multimedia Appendix 6. Most participants (24/30; 80%) reported prior exposure to the pediatric intensive care unit (ICU) or emergency medicine through at least 1 rotation. Two senior trainees (interviewee 1: female, ST6; interviewee 2: male, ST8, both ICU-experienced) took part in virtual interviews lasting 75 and 91 minutes, respectively.

Given the low participation rate and nonprobability sampling, the sample is not representative of the wider LSP trainee population, and all results should be interpreted as exploratory.

### Current Skill-Development Training

To contextualize perceptions of VR, trainees’ views on their existing training for managing critically ill children were first examined. Detailed confidence and satisfaction distributions are provided in Multimedia Appendix 7. All participants reported receiving locally organized simulation training. In addition, 16 out of 30 (53%) attended weekly local teaching, 14 out of 30 (47%) attended regional teaching, and 6 out of 30 (20%) attended college-arranged programs. Teaching formats included simulation, lectures, seminars, case-based discussions, and morbidity and mortality meetings.

In qualitative responses (n=28), lectures and seminars were valued by 5 out of 28 (17%) for building theoretical knowledge, whereas several middle-grade and senior trainees highlighted case-based discussions with senior clinicians as being particularly beneficial.

All respondents had participated in simulation training; 2 (ST5 and ST8) had acted as facilitators, and 4 (ST3, ST6 [×2], and ST8) had additionally completed advanced pediatric or neonatal life support courses (“Advanced Pediatric Life Support” or “Neonatal Life Support”). Simulation frequency varied widely between hospitals, ranging from weekly to annual sessions. In some centers, simulation was embedded into departmental teaching, with seniors covering clinical duties so juniors could attend, resulting in up to 2 to 3 sessions per month.

Participants described exposure to both low- and high-fidelity modalities. Four out of 30 (13%; ST1, ST6, ST7, and ST8) identified limited high-fidelity provision as a constraint, citing insufficient realism, particularly in representing injuries, mental status changes, seizures, respiratory effort, and other nuanced clinical signs. In contrast, 3 out of 30 (10%) emphasized the value of in situ multidisciplinary team simulation for both learning and patient safety.

Overall, simulation was viewed as an important component of developing clinical assessment, decision-making, and team-based skills, especially among junior trainees. Interviewee 2 described simulation as a “safe space” to practice and “take away what you want” without the pressure of real-life emergencies, and as helpful for building confidence. At the same time, all participants emphasized the central importance of clinical exposure, with Interviewee 1 noting, “The more you have seen, the more familiar you become, the better you get.” Repeated exposure to a broad range of presentations and nuanced cases was seen as crucial for experiential learning, pattern recognition, and confidence. In particular, both interviewees stressed that no teaching tool could fully replace real clinical experience.

Despite valuing current training, 15 out of 30 (50%) of trainees identified limited exposure to high-acuity situations as a key training gap, citing the rarity of critical events (10/30, 33%), junior training level (3/10, 30%), limited time in the ICU (2/30, 7%), and the fact that not all trainees complete a pediatric ICU rotation (1/30, 3%). A further 12 out of 30 (40%) perceived constraints arising from busy or understaffed clinical environments, which they felt restricted supervision, feedback, and opportunities for reflection. Senior trainees emphasized that clinical experience remains irreplaceable, although all agreed that simulation is essential for consolidating skills in a safe environment.

On a 5-point Likert scale, 23 out of 30 participants (77%) rated themselves as at least confident in identifying and managing a critically ill child, while 2 out of 30 participants (7%) reported no confidence (Multimedia Appendix 8). Confidence appeared descriptively associated with greater clinical and ICU experience, regular simulation exposure, and completion of “Advanced Pediatric Life Support” or “Neonatal Life Support” courses but showed no clear pattern by gender or subspecialty aspiration. Interestingly, there was no strong link between confidence and overall satisfaction with training; most participants reported neutral or satisfied ratings regardless of their confidence level (Multimedia Appendix 8).

### Familiarity With AI and VR

To contextualize attitudes toward VR, participants’ familiarity with AI and VR in health care was explored. On a 5-point Likert scale (“not at all” to “very familiar”), 18 out of 30 participants (60%) reported at least some familiarity with AI or VR in health care, while 12 out of 30 participants (40%) rated themselves as “not at all familiar.” AI was most commonly encountered in radiology (12/30, 40%), followed by educational chatbots (6/30, 20%), AI-enhanced simulators (4/30, 13%), and occasional VR use in cardiology (see Multimedia Appendix 8 for detailed familiarity and exposure data). One participant indicated a negative AI experience in a simulation laboratory, though details were lacking.

Only 30% (9/30; ST1 [×4], ST2 [×3], ST3 [×1], and ST6 [×1]) had received any formal training (minimal to substantial) related to virtual technologies in health care. This included introductory VR simulation sessions during foundation training in the East of England deanery (using Oxford Medical Simulation software) [13] and taster sessions at conferences. All 4 trainees who had used VR simulation during foundation training for advanced life support training described the experience positively, emphasizing the ease of use, high engagement, and perceived educational value, particularly for structured scenario rehearsal, clinical decision-making, and confidence-building. One participant (interviewee 2) had used a VR-empathy simulator and was impressed by the objective feedback on communication parameters, such as eye-contact and speech cadence.

Regarding confidence in using VR for medical training, 6 out of 30 participants (20%) felt “confident” or “very confident,” 12 out of 30 (40%) felt “a bit confident,” and 12 out of 30 (40%) felt “not confident at all.” Higher confidence was linked to prior exposure to VR (clinical or leisure), comfort with technology, and AI-related experiences. Professional VR exposure and familiarity were, by trend, more common among junior trainees, whereas the subgroup reporting no confidence was linked to a lack of VR exposure. This group comprised both junior and senior trainees, with a very slight predominance of more senior physicians.

An exploratory comparison indicated that trainees with prior VR experience reported higher confidence in using VR for training than those without (Mann-Whitney *U* test=86.5; *P*=.04); however, this finding remains tentative due to small subgroup sizes. Overall, only 5 out of 30 (17%) participants had used VR simulation in a medical context, and this limited exposure needs to be considered when interpreting their perceptions. These patterns underscore the interpretive limitation that most views represent expectations rather than experience-based judgments.

### Anticipated Educational Role of VR Simulation in Managing Critically Ill Children

To assess LSP trainees’ expectations and reservations regarding VR simulation, we explored their views on its potential integration for training in managing critically ill children, using 5-point Likert scales and open-ended questions. Detailed attitudinal response distributions are provided in Multimedia Appendix 9.

As most trainees had not used VR in pediatric critical care, their responses capture anticipated rather than experienced educational value. Three participants (across training levels ST1-ST5) with prior VR simulation exposure in pediatric or adult critical care described it as “very useful and engaging,” highlighting immersion, the ability to revisit scenario segments, and consistent exposure to rare cases. These perspectives illustrate how VR might support rapid decision-making and learning from immediate, simulated consequences, while also noting limitations in hands-on procedural practice and team dynamics.

On a 5-point Likert scale, 27 out of 30 (90%) trainees rated VR simulation as at least somewhat effective for training in managing critically ill children. Lack of prior VR experience did not preclude positive expectations; many VR-naive trainees still anticipated benefits. Some more experienced trainees expressed neutral or skeptical views, but positive expectations were present across all training levels. Exploratory analyses did not identify statistically significant differences in perceived overall usefulness between VR-experienced and VR-naive participants (Mann-Whitney *U* test=115.5; *P*=.69).

Participants anticipated that VR could enable infinite replication of realistic, nuanced scenarios (27/30, 90%), integrating visual and auditory cues and simulating clinical changes more effectively than mannequins. Trainees also appreciated VR’s potential for tailoring scenarios to individual needs (27/30, 90%) and delivering nuanced (28/30, 93%) and rare (28/30, 93%) cases. The immersive environment was perceived as conducive to learning from mistakes (28/30, 93%) and building confidence (25/30, 83%). Most participants assumed that VR’s flexibility and accessibility (26/30, 87%) could increase training opportunities and better accommodate busy clinical schedules. They perceived potential benefits for skills, such as task-switching, prioritization, delegation (24/30, 80%), and clinical reasoning (24/30, 80%). Many (eg, interviewees 1 and 2) emphasized its potential to deliver rare and complex cases, support individualized scenarios, and provide a psychologically safe environment to practice, make mistakes, and build confidence. Several trainees noted that VR might be particularly beneficial for more introverted learners, who may feel less comfortable in traditional group simulations.

However, expectations were not uniformly uncritical. Some participants questioned VR’s current ability to support team-based learning and real-time facilitation, and several found it difficult to envision its role in procedural or complex communication skills training.

While 28 out of 30 (93%) indicated they would be willing to try VR simulation if available, this willingness reflects openness to experimentation rather than evidence of educational effectiveness. Across all training levels, trainees generally perceived VR as potentially useful at multiple stages of training. However, irrespective of seniority or training level, they consistently anticipated the greatest benefit for junior trainees, particularly before or early in registrar-level training, when exposure to critically ill children is still limited.

Exploratory inferential analyses were conducted to examine subgroup differences. No significant differences were observed between junior (ST1-ST3) and senior (ST4-ST8) trainees regarding perceived usefulness, realism, or acceptability of VR-based simulation (all *P*>.05), suggesting broadly consistent perceptions across training levels. No statistically significant differences were observed between VR-experienced and VR-naive trainees regarding willingness to trial VR (Mann-Whitney *U* test=122.0; *P*=.51); overall, most participants expressed openness to trying VR simulation if made available.

### Perceived Barriers and Facilitators to VR Adoption

Participants identified several perceived barriers to integrating VR simulation into pediatric training. Cost was the most frequently endorsed barrier (24/30, 80%), encompassing hardware, software, maintenance, and faculty training. Cost concerns encompassed hardware acquisition, maintenance, and training expenses. Interviewee 1 questioned the feasibility of additional funding, citing the £30,000 cost (£1=US $1.34 as of June 15, 2026) of a high-fidelity simulator already shared across the hospital. Conversely, interviewee 2 anticipated decreasing costs with advancing technology. A detailed overview of perceived barriers and facilitators is provided in Multimedia Appendix 10.

Accessibility and technical infrastructure (17/30, 57%), technological literacy (17/30, 57%), and lack of familiarity and support among key stakeholders (25/30, 83%) were also highlighted. Additional concerns included time constraints (13/30, 43%), limited haptic feedback, and practical challenges such as motion sickness (1/30, 3%) and issues for glasses wearers (1/30, 3%). P3 also noted that VR currently cannot replace in-situ simulation, an important component of experiential learning.

Accessibility challenges were linked to infrastructure disparities, including unreliable WiFi and resource inequality between urban and rural areas. While 17 out of 30 patients (57%) flagged technological literacy as a barrier, Interviewee 2 noted that most current trainees are comfortable with digital tools, suggesting that adaptation to VR may be less problematic for newer cohorts.

Lack of familiarity and knowledge among stakeholders was perceived as a major barrier. Participants recommended initiatives, such as taster days, practical demonstrations, and informational sessions to increase awareness and build early buy-in from managers and trainers, which were perceived as essential for securing funding. Demonstrating VR’s educational effectiveness through research was also suggested to support adoption.

Trainees emphasized the importance of VR technology being realistic, user-friendly, easily maintained, and offering a variety of clinical scenarios. They recommended piloting VR implementation within hospitals affiliated with medical schools for initial refinement before a wider rollout.

Taken together, in this small exploratory study, pediatric trainees described simulation and clinical exposure as central to developing skills in managing critically ill children, while identifying gaps in exposure to high-acuity cases and in supervision and feedback. Familiarity with VR simulation was limited, yet most trainees anticipated that VR could act as a useful adjunct to existing training—particularly for junior physicians—if key barriers, such as cost, infrastructure, and stakeholder unfamiliarity, could be addressed. These findings represent expectations and illustrative perspectives rather than evidence of VR’s actual effectiveness and should be interpreted accordingly.

Exploratory inferential analyses were conducted to examine subgroup differences. No significant differences were observed between junior (ST1-ST3) and senior (ST4-ST8) trainees regarding perceived usefulness, realism, or acceptability of VR-based simulation (all *P*=.05), suggesting broadly consistent perceptions across training levels. Trainees with prior VR exposure reported higher confidence in using VR for medical training compared with those without previous exposure, although differences in perceived educational value did not consistently reach statistical significance.

## Discussion

### Objective Findings

This exploratory, mixed methods study examines how pediatric trainees within the LSP conceptualize and anticipate the role of VR simulation for developing skills relevant to managing critically ill children. Overall, trainees reported limited prior exposure to VR simulation in both general and medical contexts. Despite this, trainees expressed cautious optimism regarding its potential role as a supplementary training modality, particularly for junior physicians with limited exposure to high-acuity clinical scenarios. Anticipated benefits of its adoption included opportunities for repeated exposure to rare and complex situations, enhanced accessibility, and a psychologically safe environment for early skill development. At the same time, trainees identified important barriers to implementation, including cost, limited infrastructure, and lack of stakeholder familiarity, alongside facilitators to its implementation, such as taster sessions, faculty advocacy, and early engagement. Participants also highlighted limitations of current approaches to VR-simulated training, including the lack of interaction with the team and senior faculty members. These findings suggest that trainees form structured and internally consistent expectations about emerging educational technologies, even in the absence of direct experience, shaped by perceived gaps in current training and broader familiarity with digital innovation.

This study contributes to the emerging literature on VR-based simulation in postgraduate medical education by exploring pediatric trainees’ perceptions of its potential role in critical care training for recognizing and managing critically ill children. In contrast to the prevailing VR literature, this study offers insight into how postgraduate pediatric trainees conceptualize VR’s potential role within high-stakes, low-frequency emergency care [9,26]. Rather than evaluating VR’s educational effectiveness, the findings illuminate anticipated value, perceived limitations, and contextual factors shaping readiness for adoption.

Consistent with established simulation literature, simulation, particularly high-fidelity, faculty-supervised sessions, remained central to skill development across the cohort [8,27]. This aligns with extensive evidence underscoring simulation’s critical role in progressing from knowledge acquisition to safe, independent clinical practice [28]. Trainees’ reflections were consistent with experiential and reflective learning theories, highlighting the need to repeatedly apply cognitive frameworks to dynamic clinical problems [22,29]. Yet, participants emphasized persistent structural limitations: infrequent exposure to high-acuity cases, constrained access to simulation resources, and limited faculty availability for feedback and debriefing [8,11].

Junior trainees particularly valued structured simulation practice as a supplement due to limited real-world exposure. In contrast, senior trainees repeatedly stressed that some learning—particularly managing clinical complexity, navigating distressed families, coordinating multidisciplinary teams, and functioning under intense time pressure—remains intrinsically tied to context-rich real-world encounters [11,26]. These skills require rich contextual cues and emotional dynamics that current VR platforms cannot yet replicate [11]. Their emphasis complements cognitive load theory [30], which posits that foundational skills are best acquired in controlled environments, whereas complex, ill-structured tasks require authentic practice. This distinction reinforces the view, expressed across the sample, that VR is best positioned as supplementary, not substitutive.

Within this context, trainees articulated several anticipated educational advantages of VR. These included the ability to rehearse rare scenarios, repeat challenging segments, and tailor content to individual needs—features well-documented in the VR-education literature [11,12,26]. Notably, participants anticipated that VR could expand access to scenario-based learning without proportional increases in faculty time or resources. Concerns raised about teamwork and feedback reflect current technological limits but may lessen with advances in multiuser environments and automated performance metrics [31].

Gaps in frequency, diversity, and supervision suggest opportunities for VR to act as a scalable supplement, particularly for junior trainees, by providing repeatable, safe, and accessible practice to acquire foundational skills in line with cognitive load theory [30]. By shifting some training into VR, faculty capacity could be freed for more frequent high-fidelity simulations, allowing experienced trainees to practice nuanced clinical emergencies alongside real-world cases, in line with recommendations for regular skills practice to maintain proficiency [32].

Participants described a redistribution of training activities, with VR perceived as particularly suited to lower-complexity learning objectives. Specifically, VR was viewed as an effective platform for rehearsal-based learning, including early exposure to common deterioration patterns and structured decision-making within predictable clinical constraints, supporting early skill acquisition while managing cognitive load. This redistribution was expected to free faculty time for in-person, high-fidelity simulation sessions focused on higher-order learning outcomes that require nuanced feedback, rich interpersonal interaction, and the development of complex procedural and communication skills. This “tiered simulation ecosystem” has been proposed in prior research but remains underexplored within pediatrics; our findings provide early qualitative support for its perceived feasibility.

The exploratory inferential findings provide further nuance to these perspectives. VR-experienced trainees reported higher confidence in using VR for training than their VR-naive peers, and VR familiarity correlated positively with VR confidence. This pattern aligns with technology acceptance literature, suggesting that familiarity—rather than clinical competence—shapes perceived readiness for VR adoption. Although based on small subgroup numbers, these findings support the interpretive framing that attitudes toward VR reflect expectations and experiential comfort rather than demonstrated educational impact.

Perceptions were strongly shaped by prior exposure. Trainees with VR experience—particularly those who encountered VR during foundation programs or conferences—expressed greater confidence and more concrete expectations regarding its usage. Conversely, unfamiliar trainees appeared to struggle to visualize VR’s functionality, resulting in more tentative or abstract responses. This echoes technology acceptance literature [20], which demonstrates that perceived usefulness and ease of use are directly influenced by lived experience and previous work [33,34]. Importantly, demographic variables showed little influence; instead, familiarity was the dominant driver of positive attitudes. Participants’ reflections suggest that structured introductory sessions and early familiarization may be essential for accurate appraisal and subsequent adoption.

Across training levels, participants agreed that VR would be most beneficial for junior trainees. Senior trainees described how VR might scaffold early exposure, build foundational decision-making skills, and alleviate some of the anxiety surrounding first encounters with critically unwell children. Their anticipation that VR could better prepare juniors—by offering graded exposure before entering real emergencies—reflects the “cognitive apprenticeship” model [35] and mirrors pediatric educators’ long-standing desire for graduated autonomy pathways. Although these interpretations represent expectations rather than measured outcomes, they highlight a perceived role for VR in strengthening early preparedness.

Despite broad enthusiasm, trainees identified several challenges. Anticipated barriers, such as cost, infrastructure, device availability, stakeholder unfamiliarity, and inconsistent WiFi access, align with previous VR-implementation studies [36,37]. Concerns surrounding personalized feedback and team training reflect the technical constraints of current single-user VR systems and the cognitive complexity inherent in generating high-quality automated debriefing. Encouragingly, participants proposed practical enablers—taster sessions, faculty training, dissemination of evidence, and phased rollout supported by leadership and resources—consistent with recommendations from diffusion-of-innovation frameworks [26].

### Implications for Practice

Findings suggest that VR could serve as a scalable adjunct within a blended pediatric critical care curriculum. A phased approach, initially introducing VR to junior trainees, may be particularly valuable. Pilot implementation at hospitals already using VR in undergraduate education could provide a feasible starting point. Collaboration with VR-experienced deaneries, such as the East of England, may further support curriculum design and implementation.

VR’s consistency, repeatability, and on-demand accessibility align well with early-stage learning needs and could enhance readiness before clinical exposure. Importantly, integration must be framed as complementary: VR may support cognitive skill acquisition, situational rehearsal, and structured exposure to rare presentations, while traditional simulation should remain the standard for procedural skills, complex communication, and emotionally charged scenarios.

VR adoption may also allow for more efficient use of faculty capacity. By shifting foundational or repetitive practice into VR, training programs may be able to prioritize faculty-led, high-fidelity simulation for the nuanced emergency scenarios that demand expert oversight, particularly those characterized by interpersonal complexity, advanced clinical decision-making, and high-stakes procedural or communication challenges. Trainees themselves have suggested this redistribution model, and future curriculum designers may consider structured, tiered pathways aligned with learning complexity.

Participants’ reflections also highlight a broader educational opportunity: VR may help empower trainees to more actively articulate their training needs. Because VR can provide private, low-stakes rehearsal environments, junior trainees—especially those who are less confident in speaking up—may engage more regularly with deliberate practice. Over time, this could support more learner-driven training cultures and provide programs to actively solicit and respond to user feedback.

Lastly, taster sessions and active faculty engagement are likely to be essential for building familiarity, advocacy, and institutional support.

### Strengths and Limitations

This study provides early insight into how pediatric trainees conceptualize VR’s role within critical care training. Strengths include the mixed methods design, content-validated instruments, integration of theoretical frameworks, and the inclusion of trainees from across a diverse, heterogeneous deanery covering numerous pediatric subspecialties, enhancing the relevance of the findings for real-world educational settings. Furthermore, the study captures anticipatory perceptions and experiences prior to widespread implementation, offering valuable insights into how VR-based simulation may be received and integrated into training programs.

However, several limitations must be acknowledged. The participation rate was low, reflecting challenges typical in postgraduate populations and consistent with documented “survey fatigue” in health care settings [38]. While distribution occurred through an established communication channel, recruitment relied on indirect dissemination and lacked opportunities for direct personal engagement—an approach known to reduce participation rates compared with in-person invitations or repeated face-to-face reminders. The sample may therefore represent trainees with a stronger interest or more polarized views, limiting representativeness. The modest response rate also introduces the potential for nonresponse and self-selection bias, with trainees who have a particular interest in simulation, digital technologies, or educational innovation being more likely to engage. Accordingly, findings should be interpreted as exploratory and context-bound rather than statistically generalizable to the wider trainee population.

Item-level analyses indicated ceiling effects consistent with educational satisfaction research, and results should, therefore, be interpreted with appropriate caution. The presence of ceiling effects in attitudinal items and floor-pattern clustering in several barrier-related items suggests that the instrument captured strong directional views with limited response variability. While expected in perception-focused exploratory studies, these patterns likely reduced discriminative capacity and should be addressed in future instrument refinement.

Although participants represented a wide range of clinical stages, the study was limited to a single deanery. While the LSP encompasses a large and diverse population, geographical and institutional variation across the United Kingdom may influence trainees’ familiarity with simulation modalities, clinical exposure opportunities, and technological adoption. Logistical constraints prevented expansion to additional deaneries within the study period, which may have reduced the breadth of perspectives captured. Future research should, therefore, use multicenter designs spanning metropolitan and nonmetropolitan settings to mitigate potential contextual bias.

A further limitation concerns the interpretive nature of perceptions without direct VR experience. Most participants had never used VR in a clinical training context, which limits the interpretability of their views and underscores that the findings represent anticipated rather than experienced value. While this limits conclusions regarding effectiveness, it also reflects the current stage of VR implementation in postgraduate pediatric training. Comparisons between trainees with and without VR experience were constrained by small subgroup sizes, reinforcing the need for prior exposure to VR-based training sessions in future evaluations.

Finally, while the instruments underwent expert review and demonstrated strong internal consistency (Cronbach α=0.80), the study was not designed as a psychometric validation. Item-level analyses indicated ceiling effects consistent with educational satisfaction research, and results should, therefore, be interpreted with appropriate caution.

### Future Directions

Building on participants’ insights and reviewers’ recommendations, several avenues warrant exploration:

- Large-scale, multideanery studies assess perceptions, usability, and learning outcomes across varied clinical environments to address potential geographical bias.
- Feasibility studies evaluate tiered simulation models in which VR absorbs foundational training tasks, while faculty-led simulation focuses on high-complexity scenarios.
- Comparative studies between trainees with and without VR exposure aim to clarify how familiarity shapes adoption, perceived realism, and confidence.
- Longitudinal evaluations examine whether VR enhances preparedness for real clinical encounters and supports competence progression over time, while avoiding overclaiming effectiveness until evidence is established.
- Implementation science approaches investigate organizational readiness, cost-benefit considerations, and faculty training needs.
- Research on trainee empowerment, including how VR-enabled self-directed practice may support more equitable access to learning opportunities.

### Conclusions

This exploratory study highlights pediatric trainees’ cautious optimism toward VR simulation as an adjunct to existing training for recognizing and managing critically ill children. Although most participants lacked direct VR experience, they anticipated that VR could enhance preparedness, particularly among junior trainees, by providing repeatable, accessible exposure to rare scenarios. They also recognized VR’s current limitations—especially regarding procedural tasks, nuanced communication, and team dynamics. In addition, they emphasized the irreplaceability of real clinical experience and high-fidelity, faculty-led simulation.

Barriers such as cost, infrastructure, and limited stakeholder familiarity must be addressed through phased implementation, evidence generation of educational impact, and structured introductory exposure, such as taster sessions. Prior exposure emerged as a key factor shaping acceptance, underscoring the need for early opportunities to engage with VR. VR should not be considered a replacement but rather a complementary component within a blended simulation ecosystem. With careful integration, inclusive design, and equitable access strategies, VR simulation promises the potential to expand opportunities for deliberate practice, support junior trainee preparedness, and optimize faculty resources within pediatric critical care education, better preparing clinicians for complex clinical environments.

## Supplementary material

10.2196/85743 Multimedia Appendix 1 Recruitment message.

10.2196/85743 Multimedia Appendix 2 Full survey questionnaire.

10.2196/85743 Multimedia Appendix 3 Participant information sheet and consent form.

10.2196/85743 Multimedia Appendix 4 Semistructured interview guide.

10.2196/85743 Multimedia Appendix 5 Detailed psychometric evaluation of survey instrument.

10.2196/85743 Multimedia Appendix 6 Participants’ characteristics.

10.2196/85743 Multimedia Appendix 7 Trainee perceptions of current approaches to skill development for recognizing and managing critically ill children.

10.2196/85743 Multimedia Appendix 8 Knowledge and familiarity with artificial intelligence and virtual reality among London School of Paediatrics trainees.

10.2196/85743 Multimedia Appendix 9 Detailed attitudinal response distributions regarding trainees’ perceptions of the educational role of virtual reality simulation in pediatric critical care training.

10.2196/85743 Multimedia Appendix 10 Perceived barriers and facilitators to the use and implementation of virtual reality simulation for pediatric critical care skill development.

10.2196/85743 Checklist 1 CHERRIES checklist.

## Abbreviations

AI: artificial intelligence
CHERRIES: Checklist for Reporting Results of Internet E-Surveys
ICU: intensive care unit
LSP: London School of Paediatrics
ST: specialty trainee
VR: virtual reality
