# Supplementary material for: Virtual Reality Simulation in Postgraduate Pediatric Critical Care Training Based on Trainee Perceptions in London: Exploratory Mixed Methods Study
Source: JMIR Form Res. 2026 Jun 25;10:e85743. doi: 10.2196/85743 (PMC13296495; doi:10.2196/85743)
Supplement: Multimedia Appendix 1 [file formative-v10-e85743-s001.docx]

**Multimedia Appendix 1. Recruitment message**

Dear fellow trainee,

I invite you to participate in an exciting medical education project exploring the role of Virtual Reality (VR) 's role in postgraduate paediatric training. In the context of my MedEd Master's study, I am eager to understand your perspectives regarding **VR-based simulation training**. This project aims to investigate how VR can be utilised to assist us in enhancing the skills needed in identifying and **managing an acutely unwell child**.

VR-based simulation represents a **promising, supplementing training modality to traditional high-fidelity simulation**. Here users can perform history-taking, examinations, investigations, diagnoses, and treatments across a diverse spectrum of diseases and complexities using a headset only and from wherever they feel comfortable. Sim- sessions can be undertaken either independently or as part of a group session. Users will also receive **individualised feedback** after each scenario based on their performance to help improve their practice. **To participate in the study (survey or interview), you don't need any previous experience with VR-based simulation training!**

The study involves completing a **brief online survey**, which should take **approximately 10 minutes** to complete. Once you have submitted the questionnaire, you will not be able to withdraw your answers and so please ensure you are happy with your responses prior to submission.

Additionally, you can participate in **a virtual interview**, lasting **approximately 30min**s. If you are interested in participating in the interview then please register your interest using the link at the end of the survey. **Participation** is entirely **voluntary** and will have **no impact on your trainin**g. Rest assured, your responses will be anonymised.

**Your input would be invaluable and help advance medical education!**

**Thank you!!**
