# Supplementary figures and images for: Virtual Reality Simulation in Postgraduate Pediatric Critical Care Training Based on Trainee Perceptions in London: Exploratory Mixed Methods Study
Source: JMIR Form Res. 2026 Jun 25;10:e85743. doi: 10.2196/85743 (PMC13296495; doi:10.2196/85743)

**Multimedia Appendix 2. Full survey questionnaire**


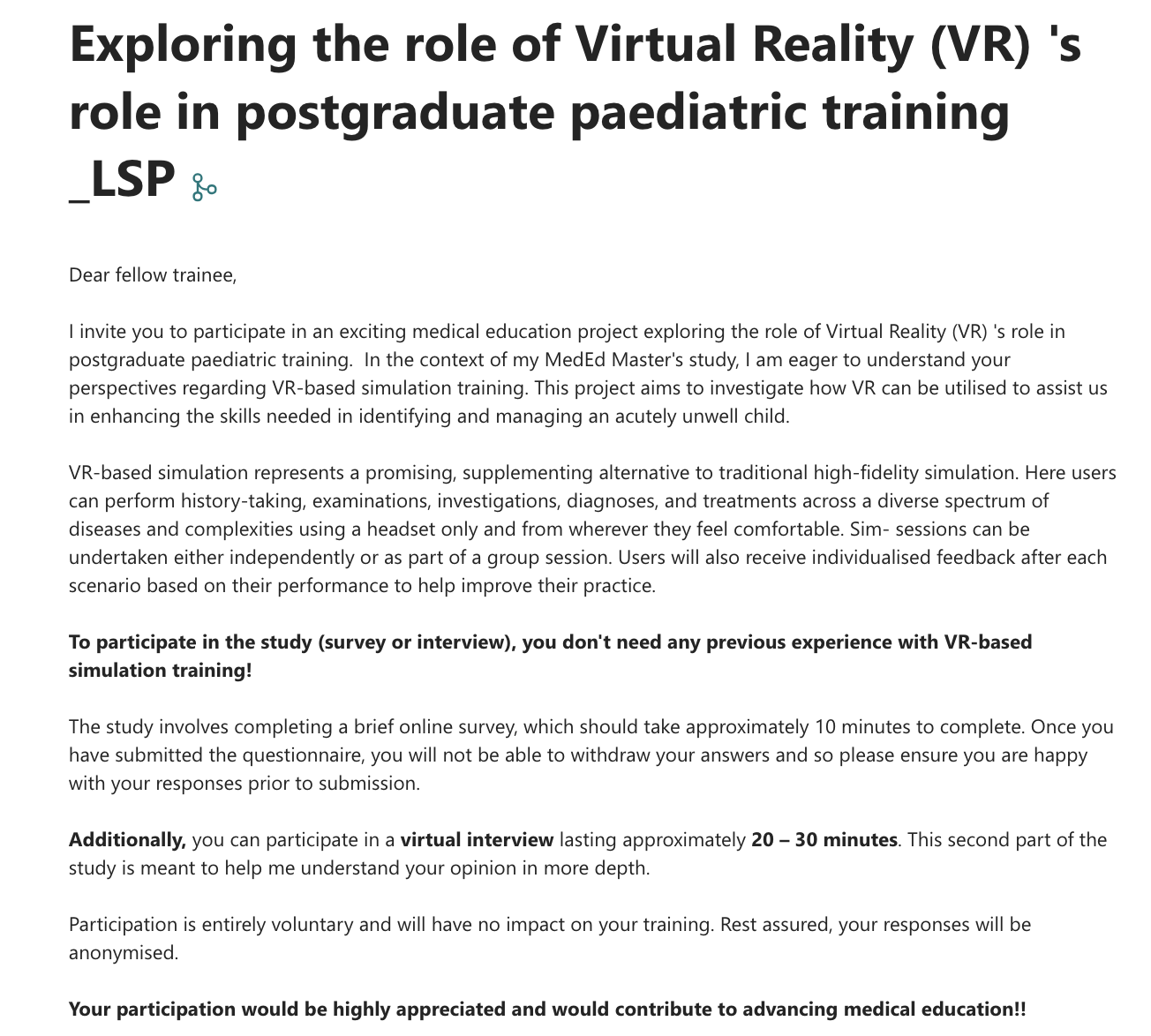


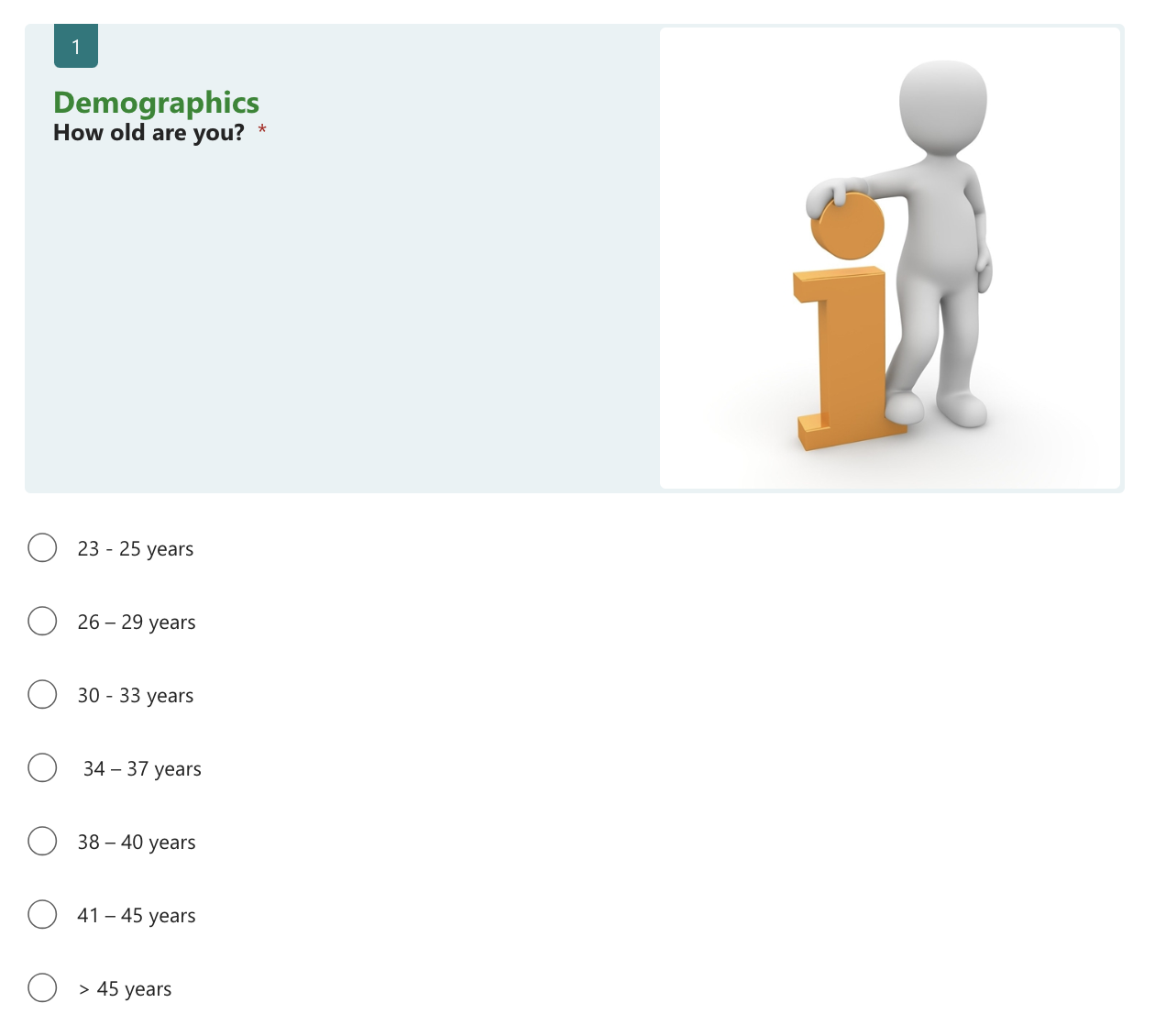


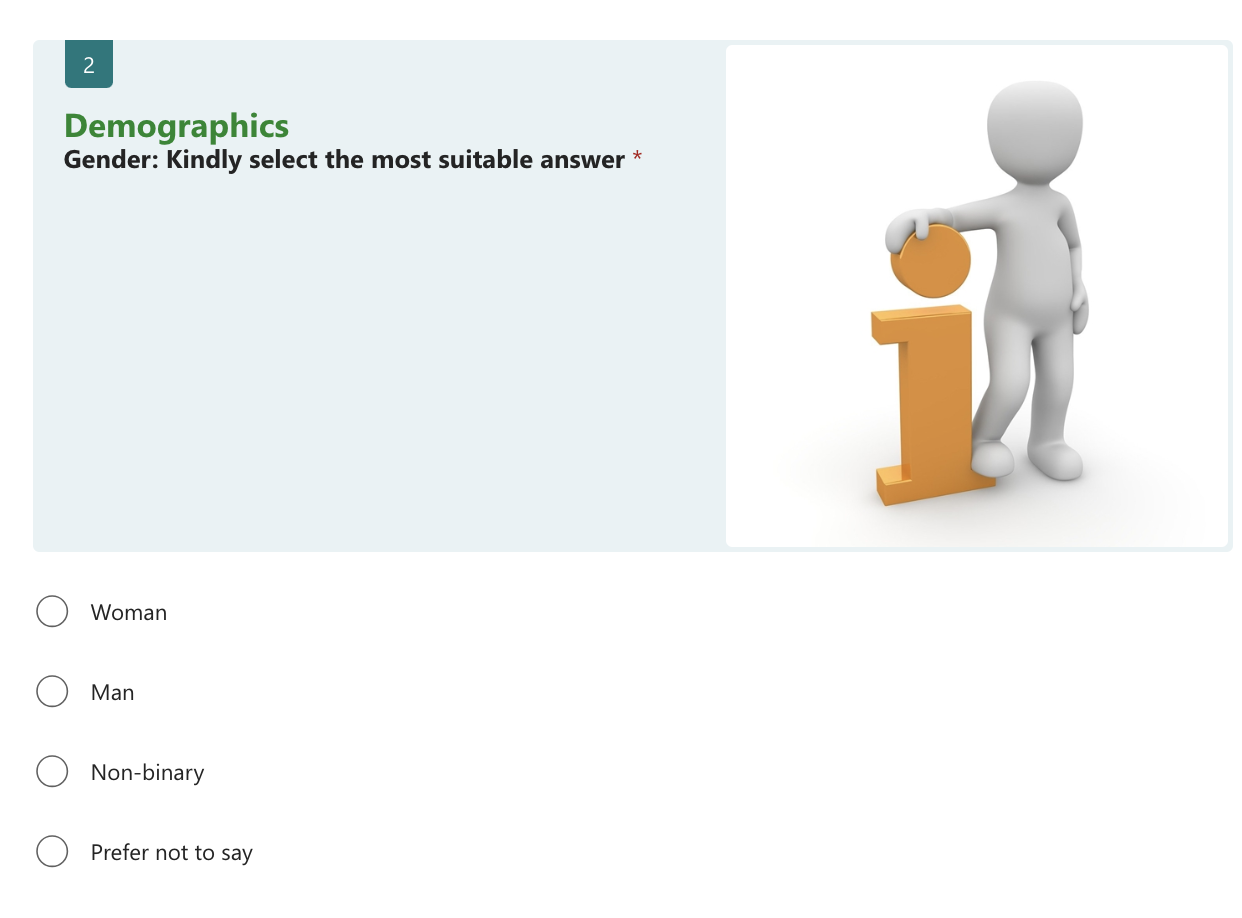


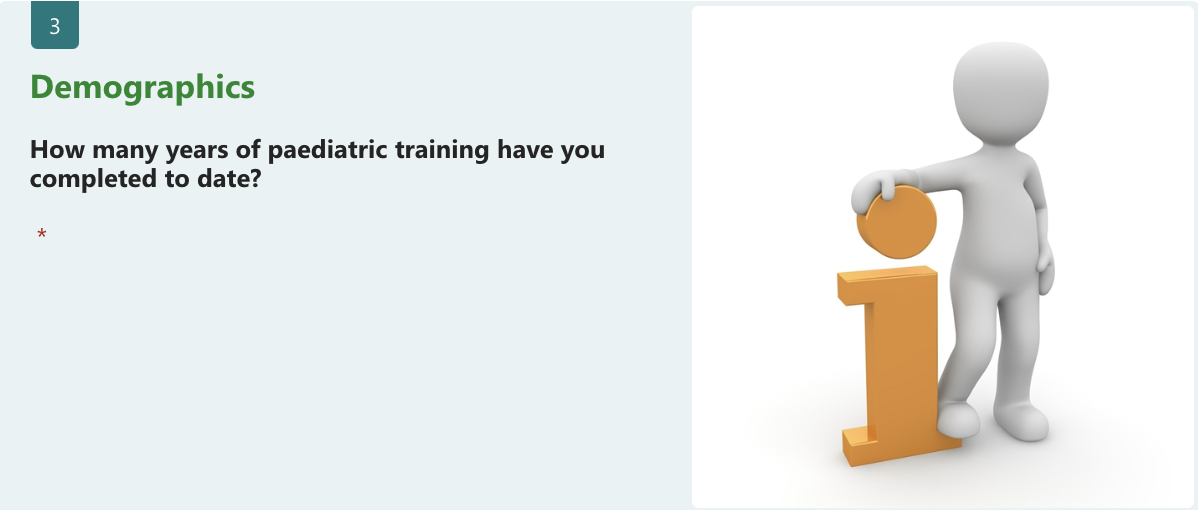


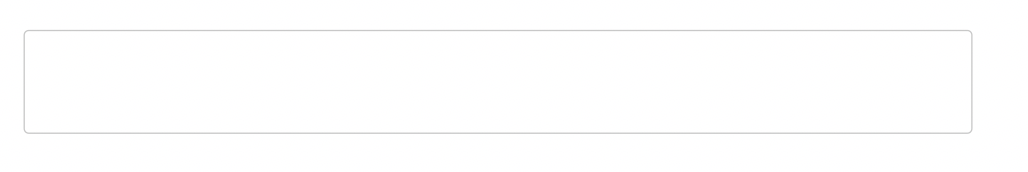


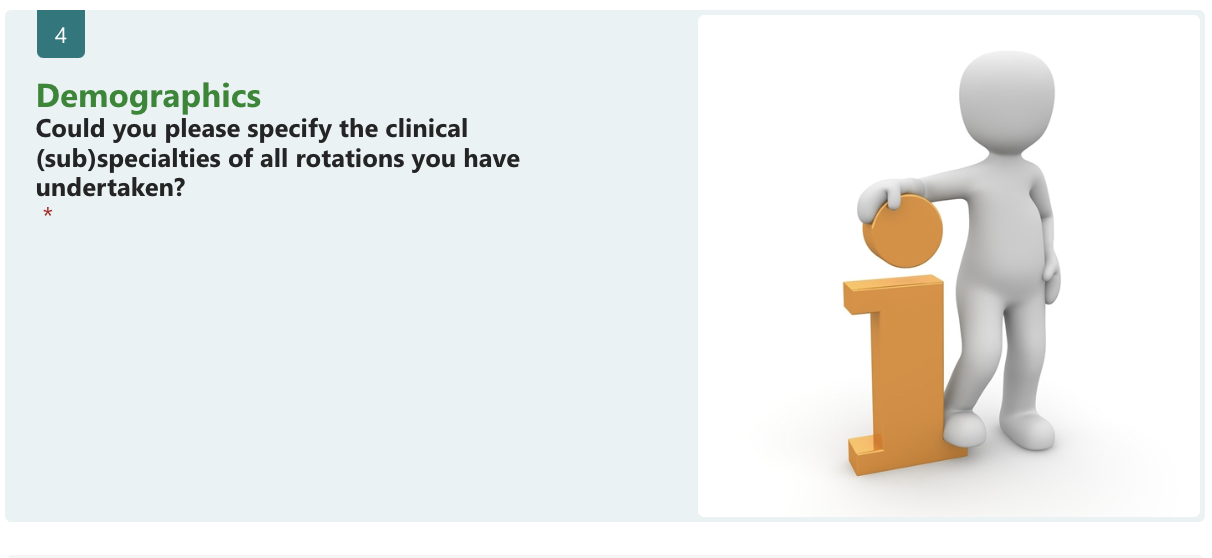


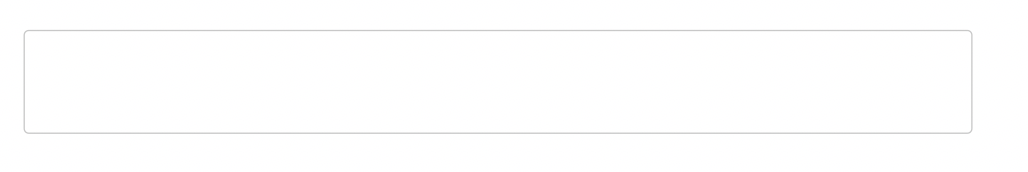


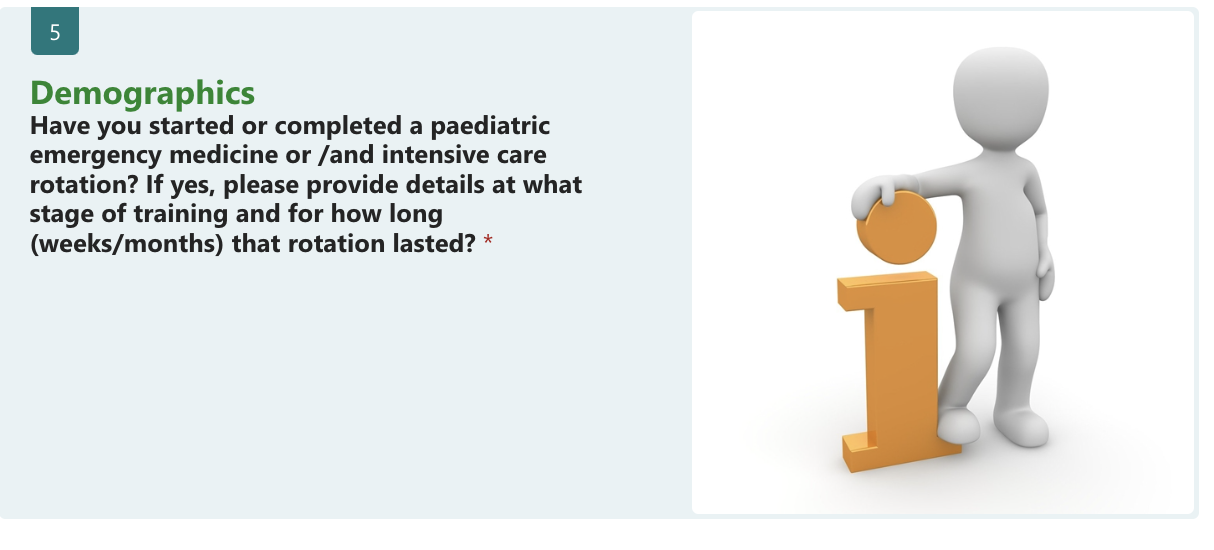

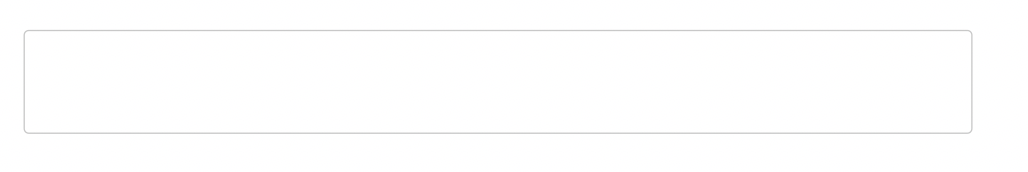


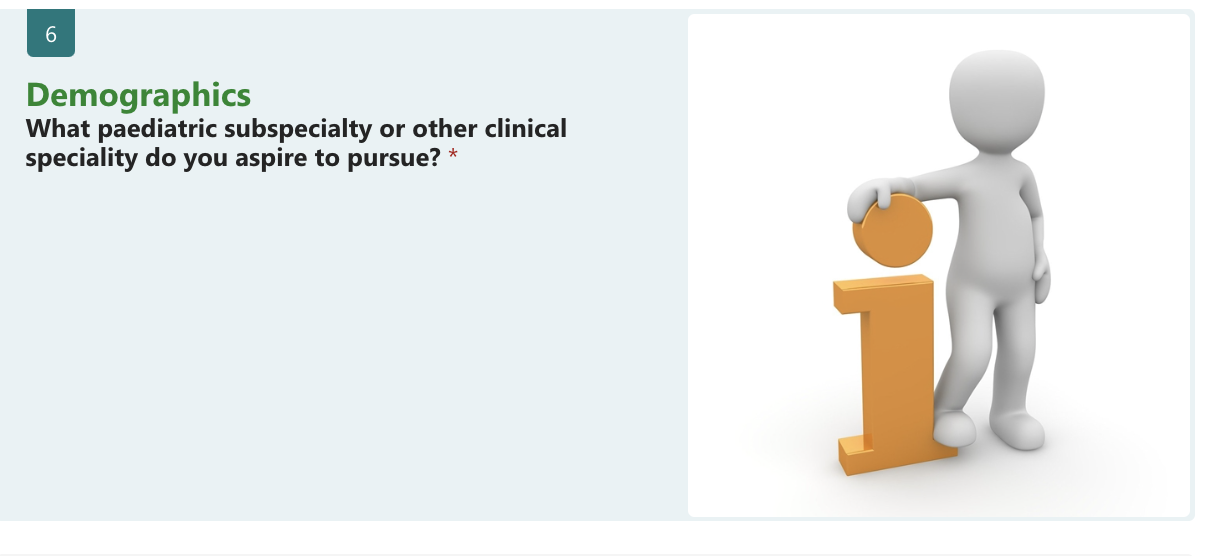


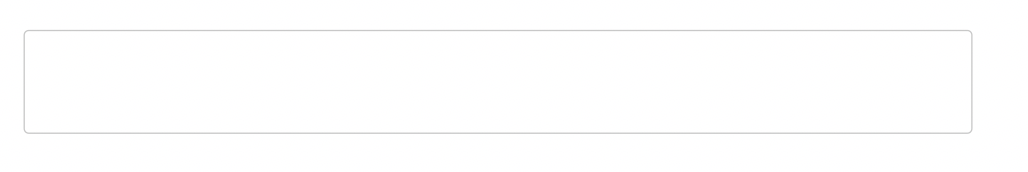


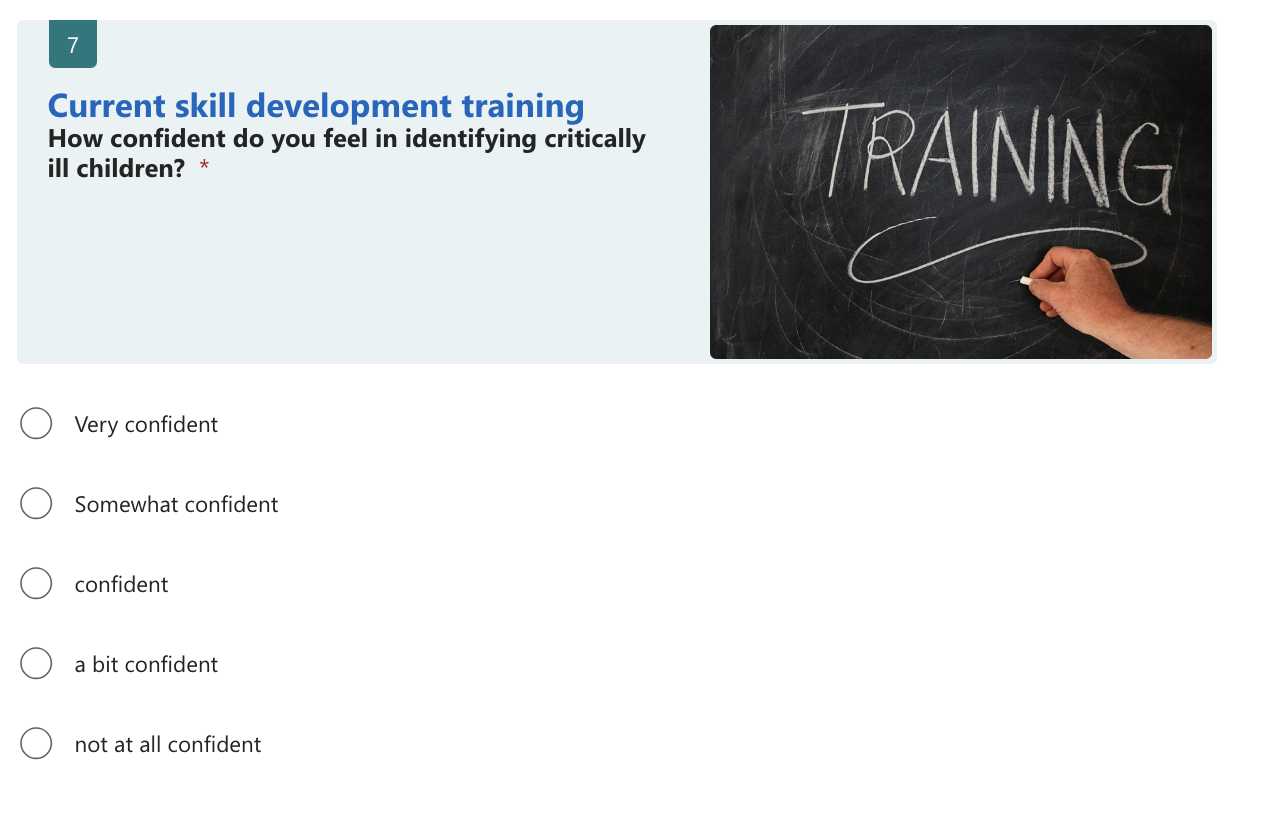


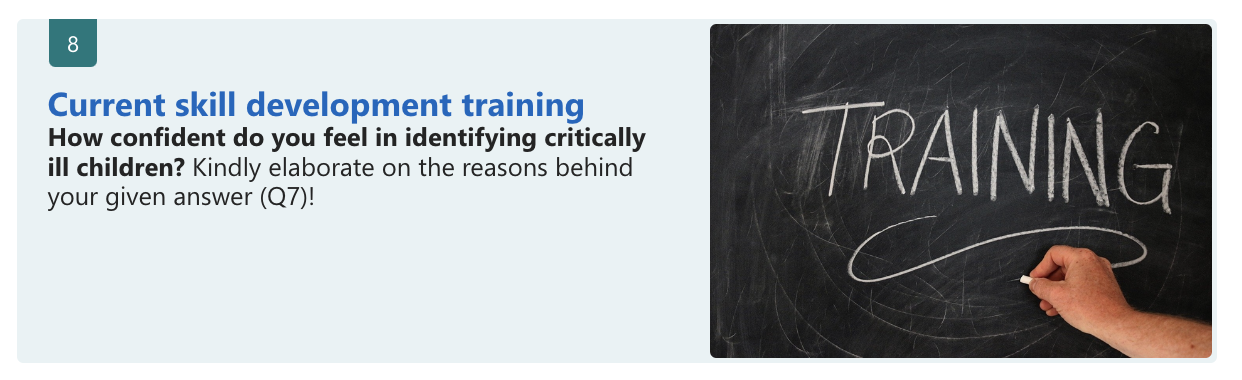

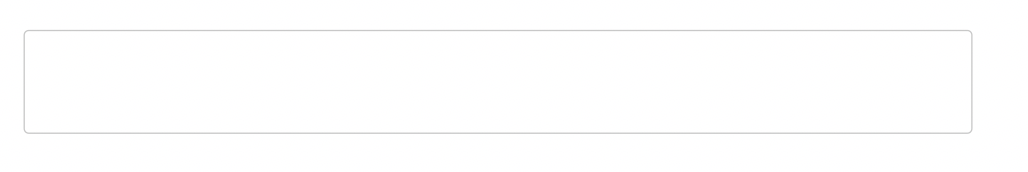


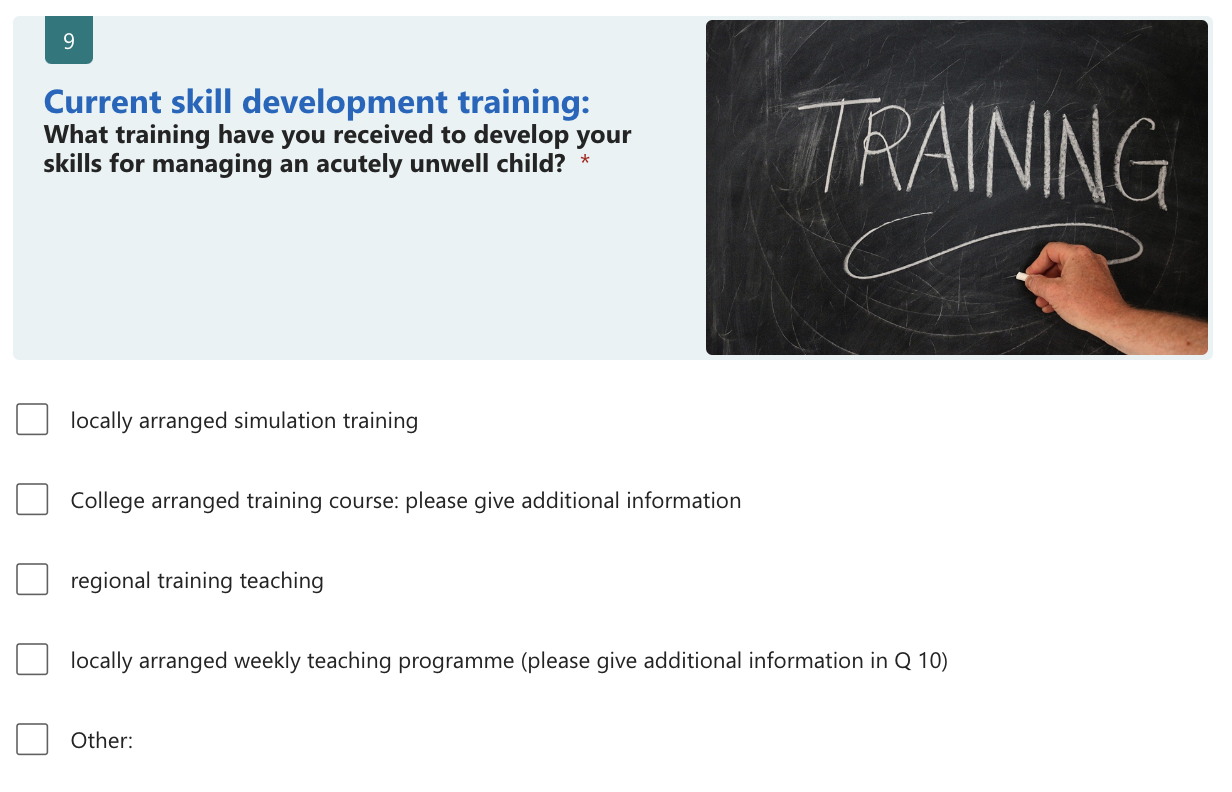


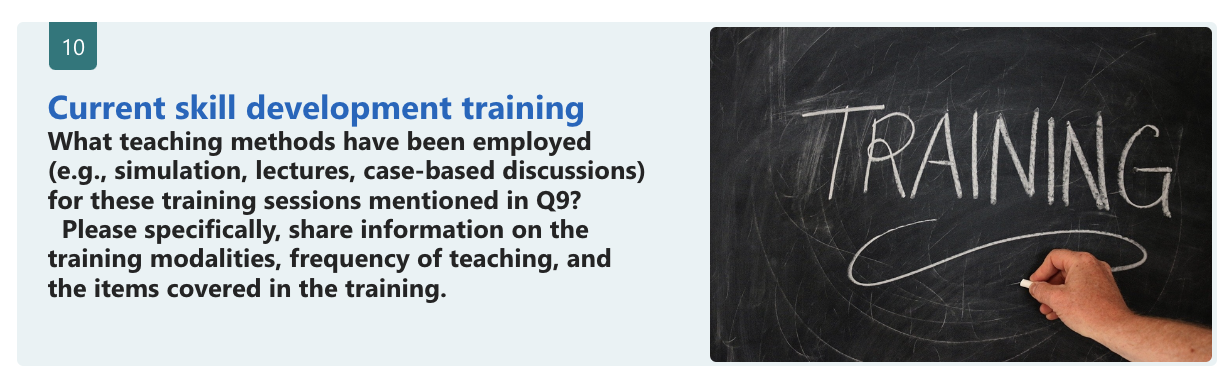

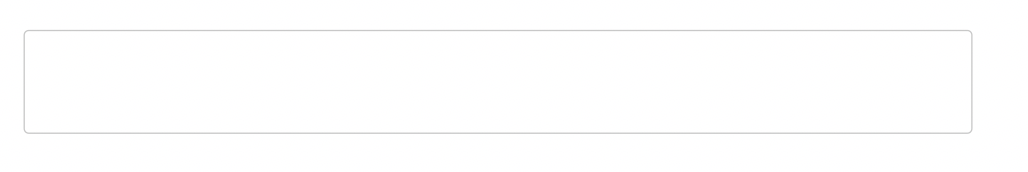


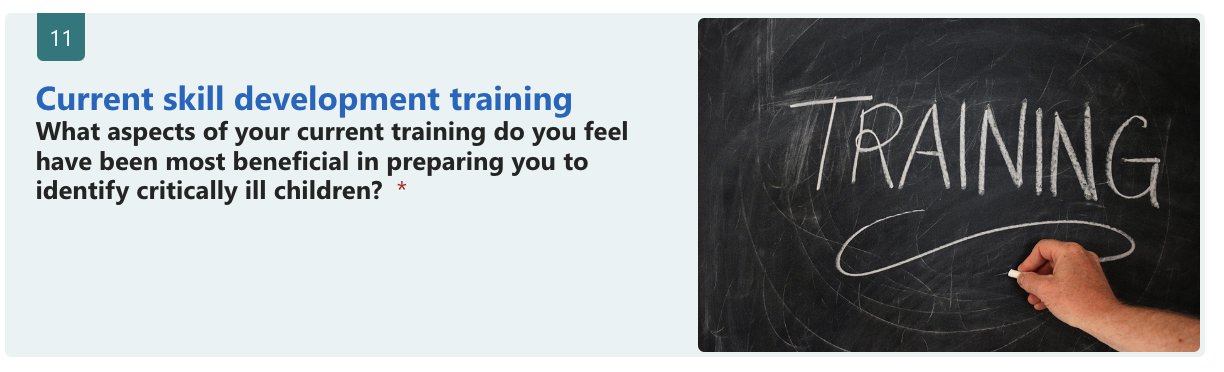

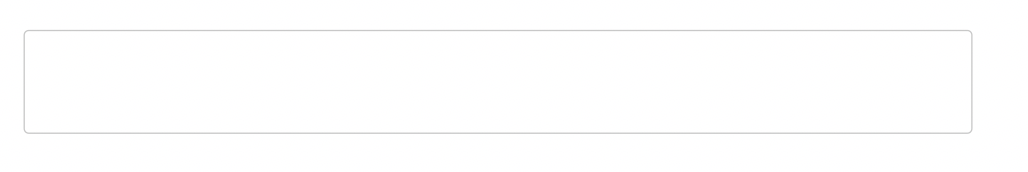


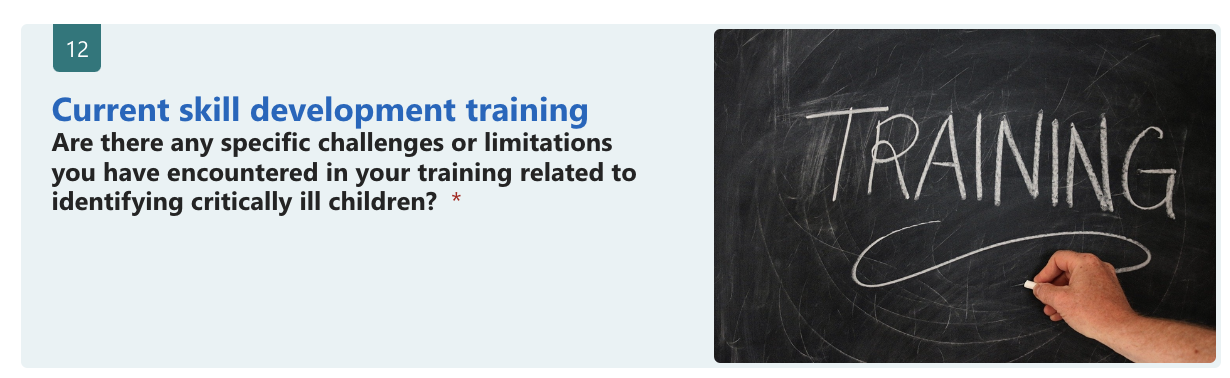

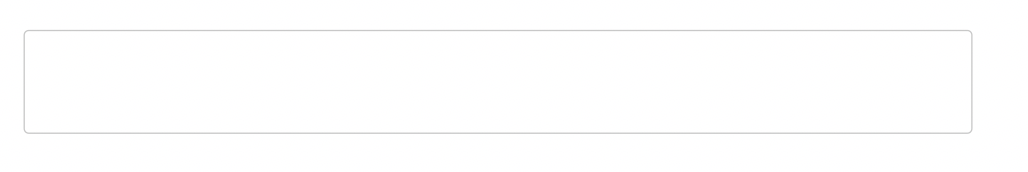


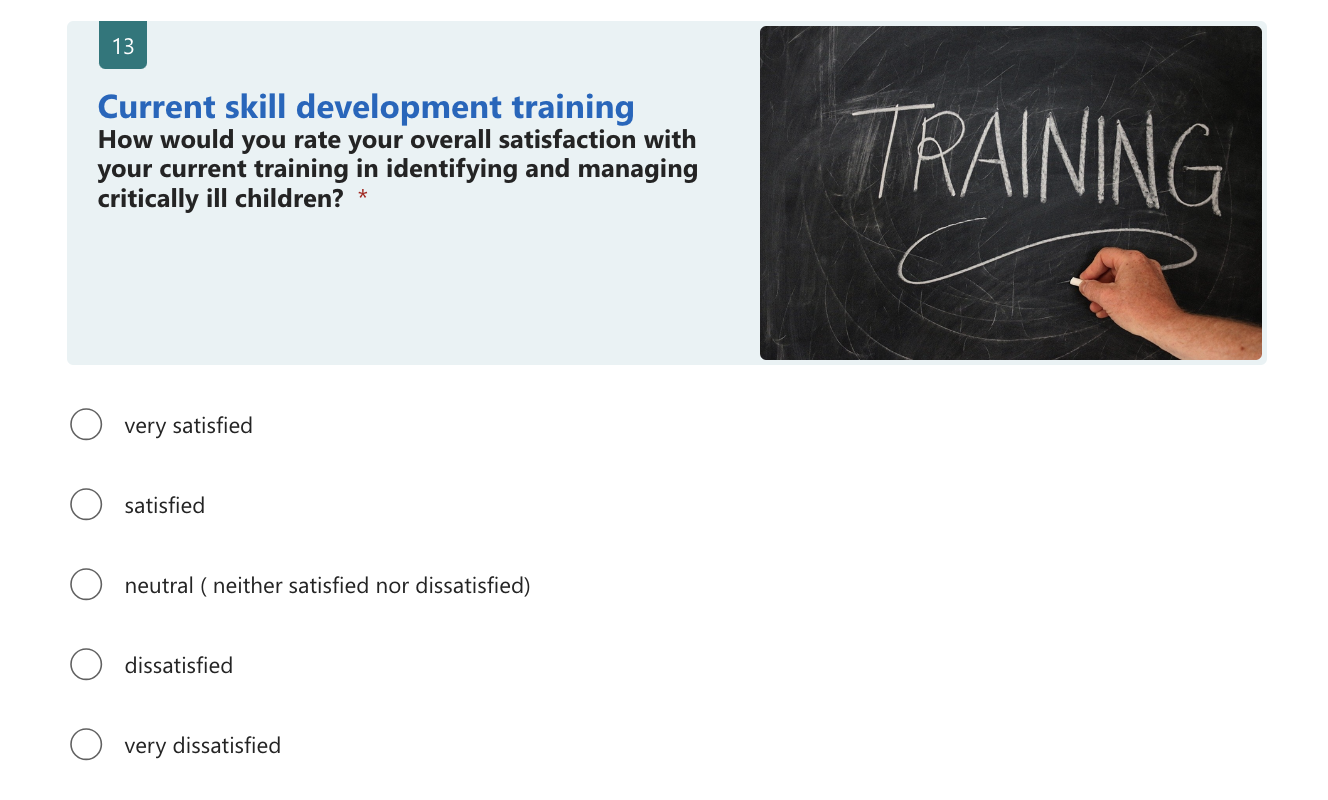


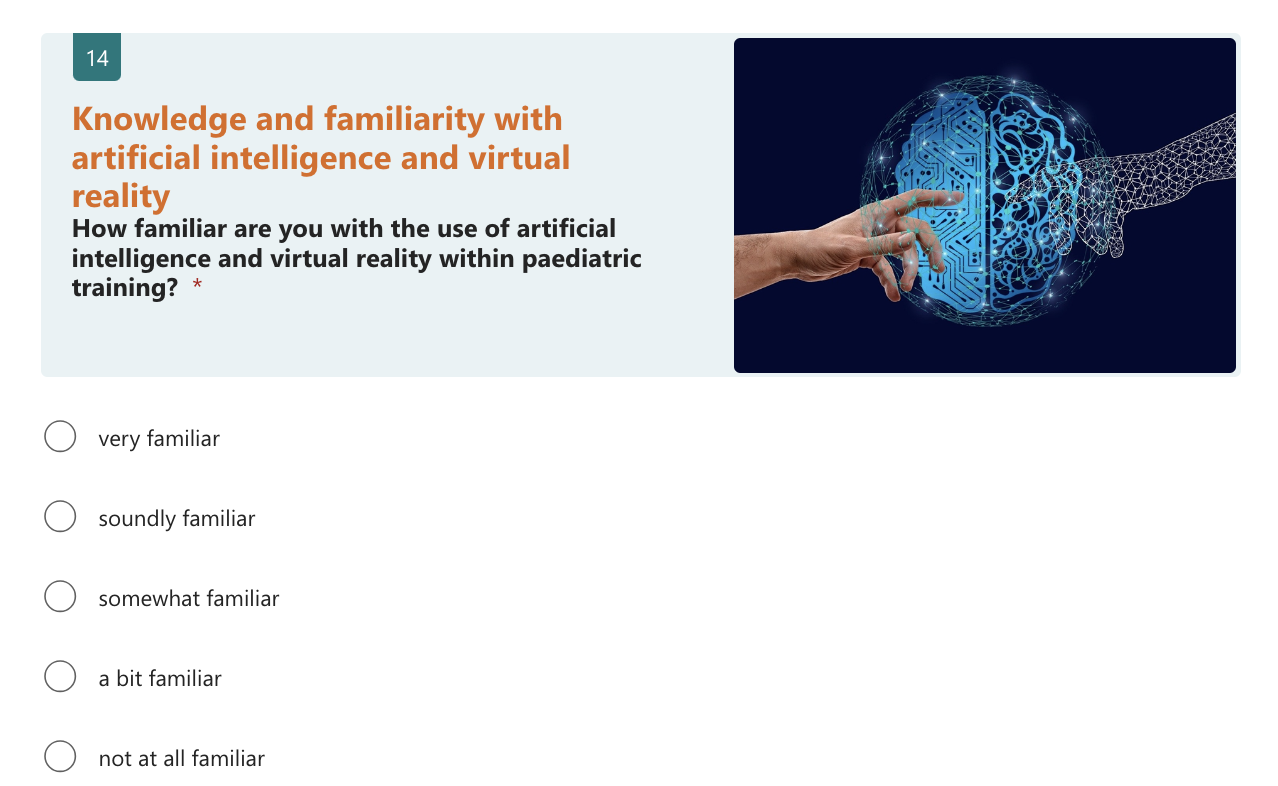


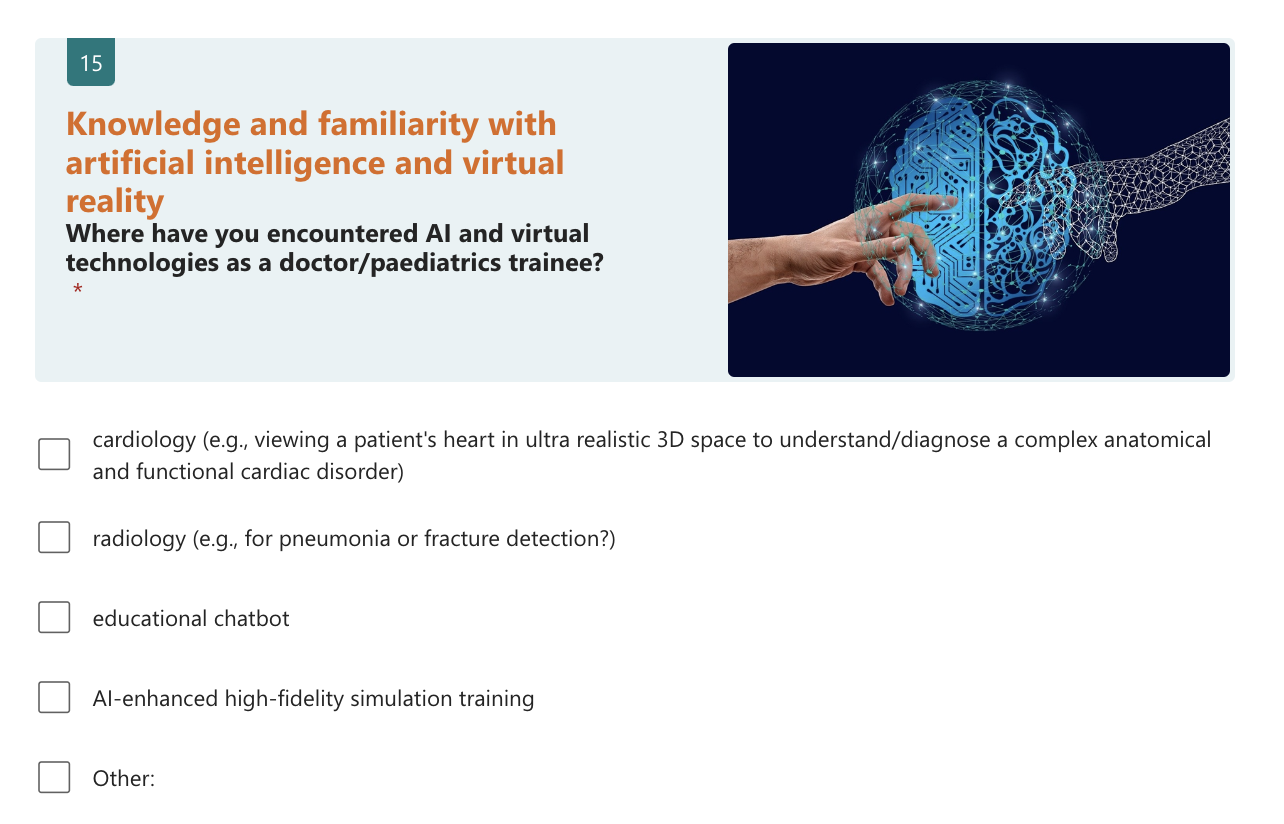


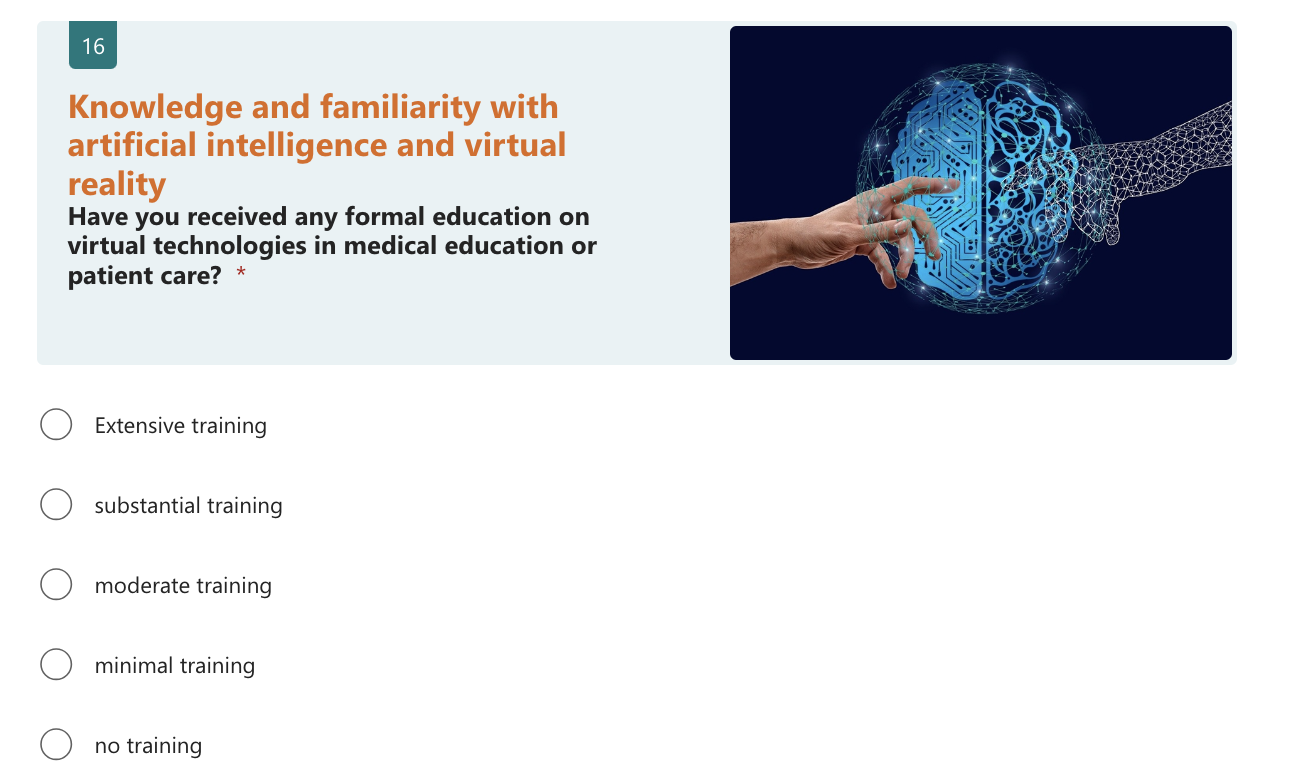


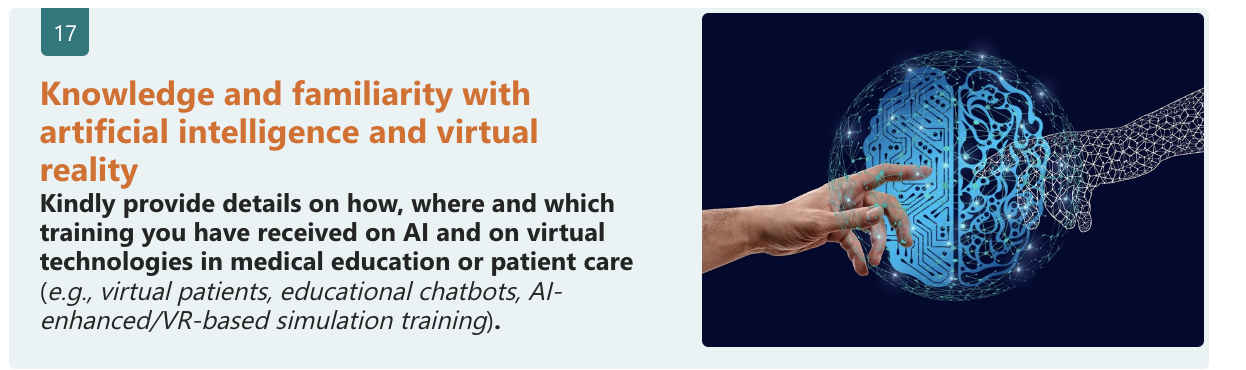

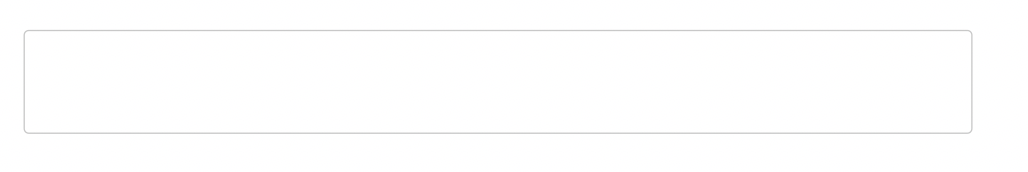


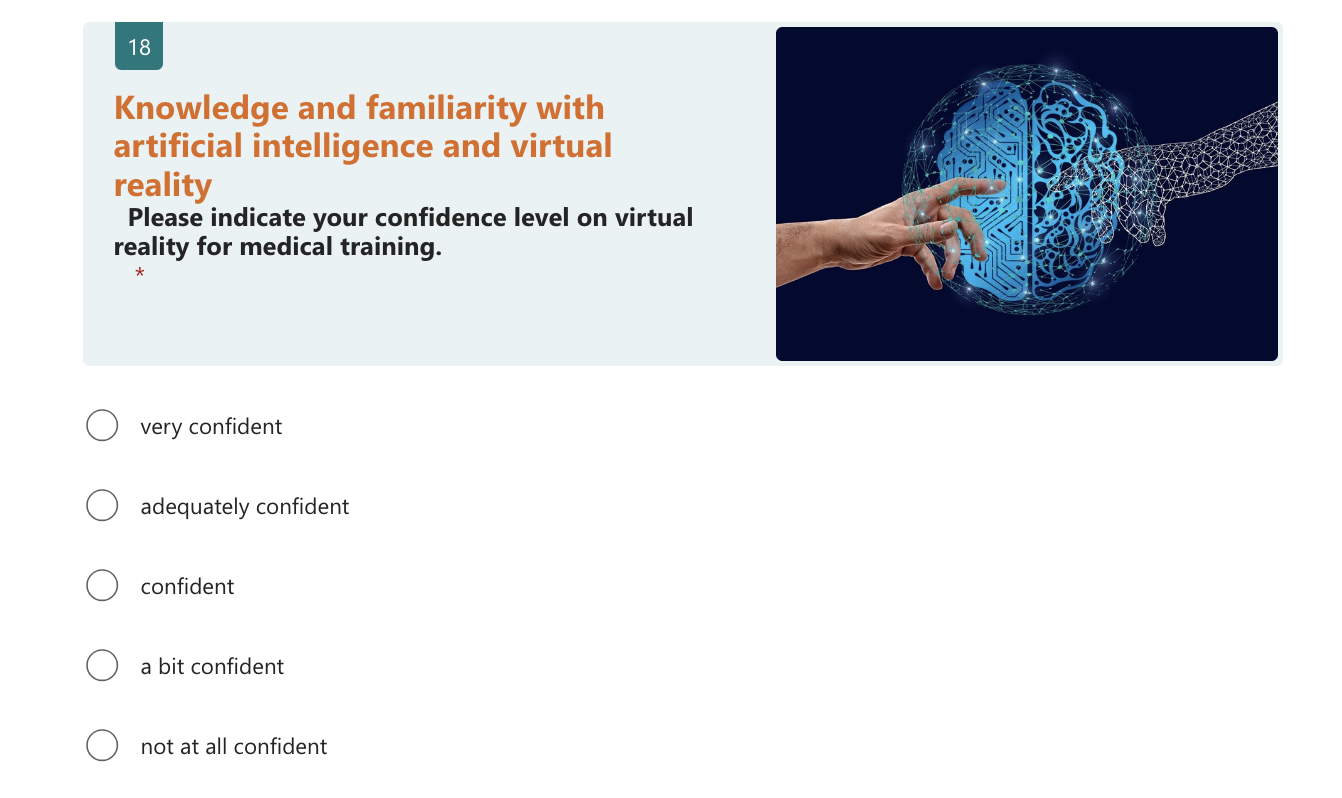


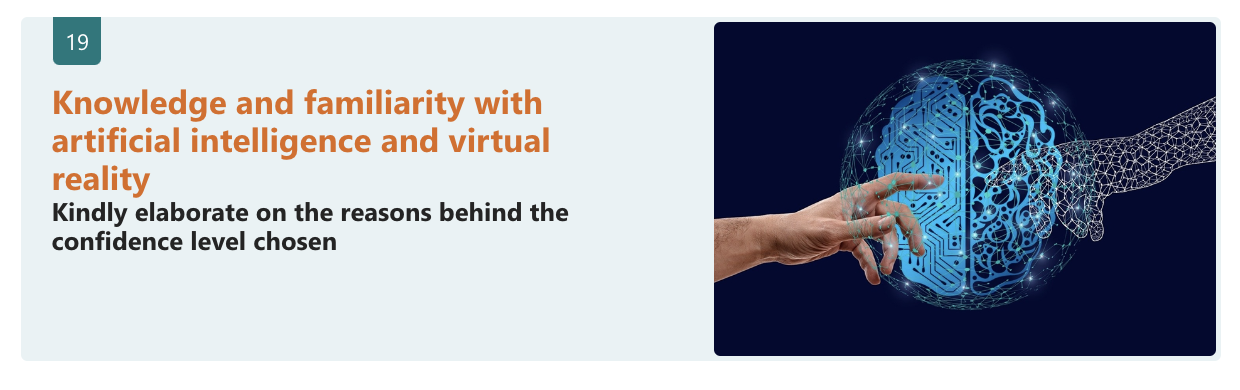

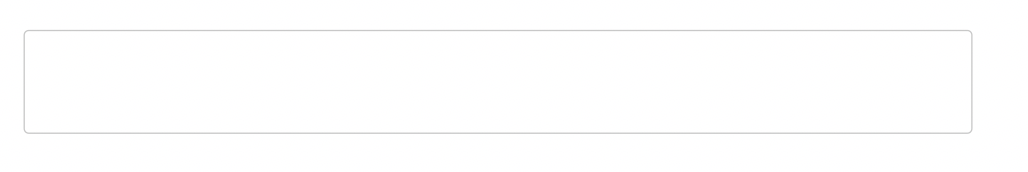


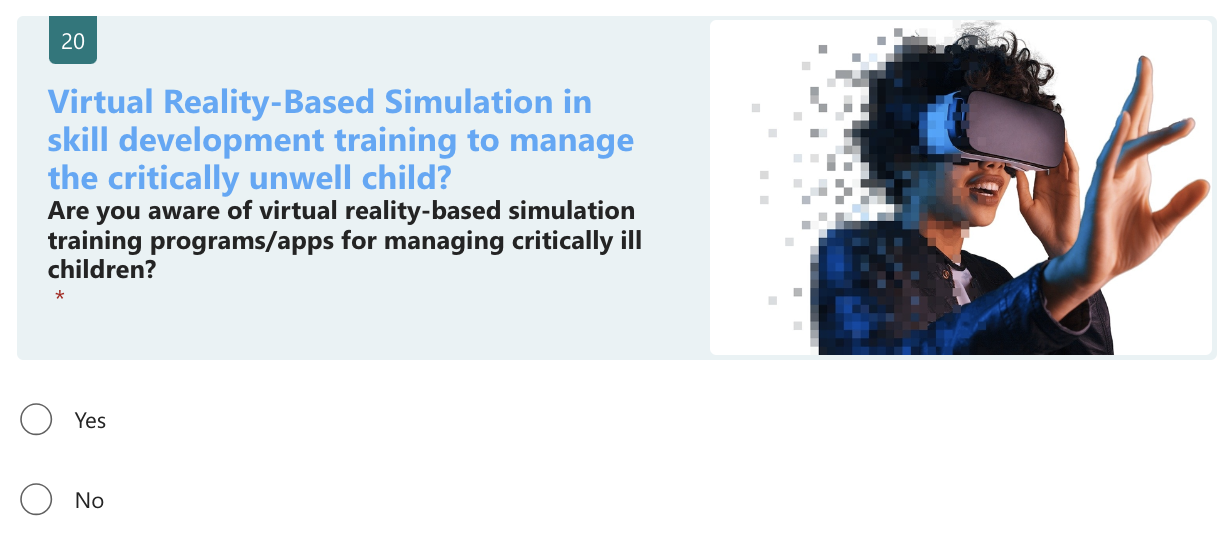


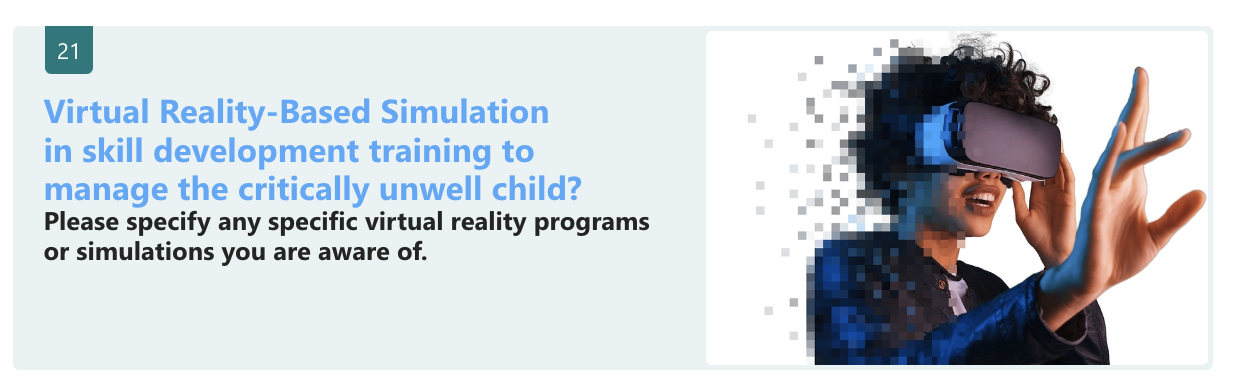

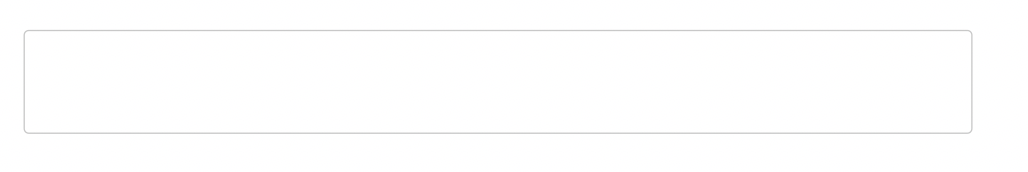


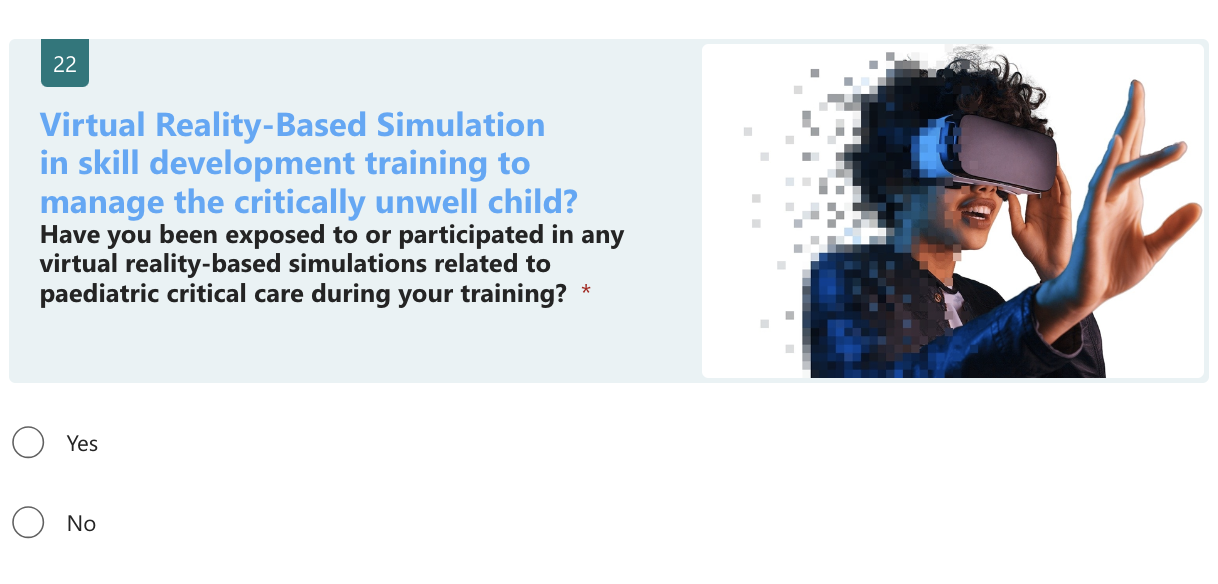


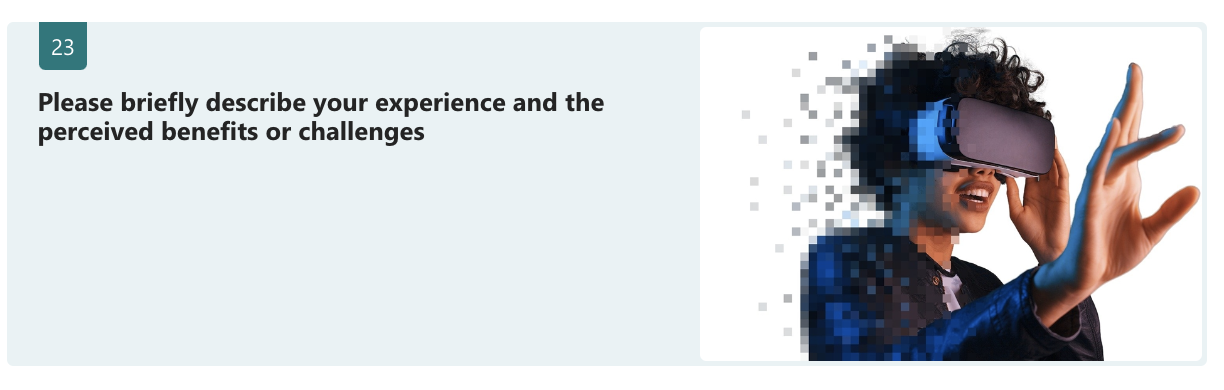


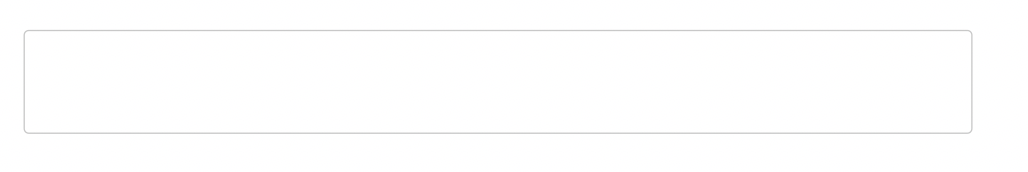


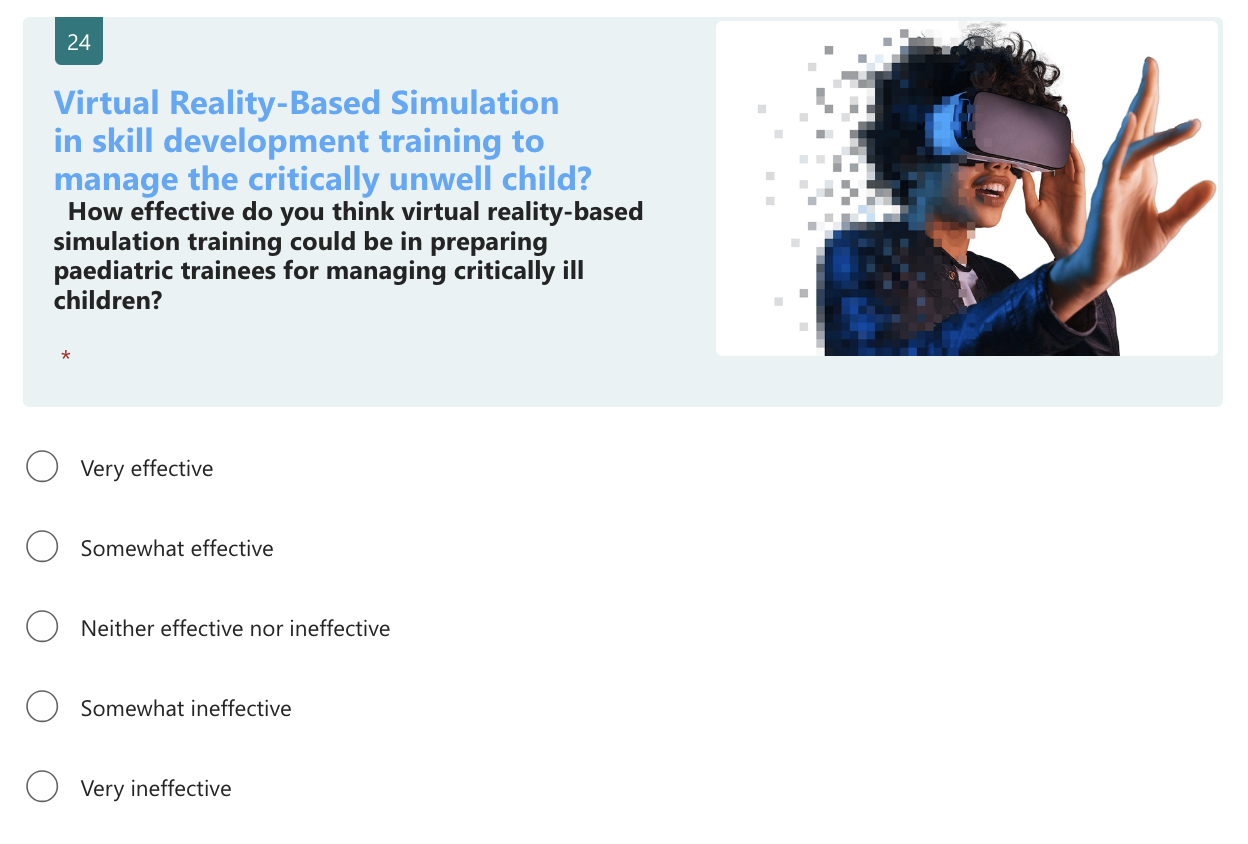


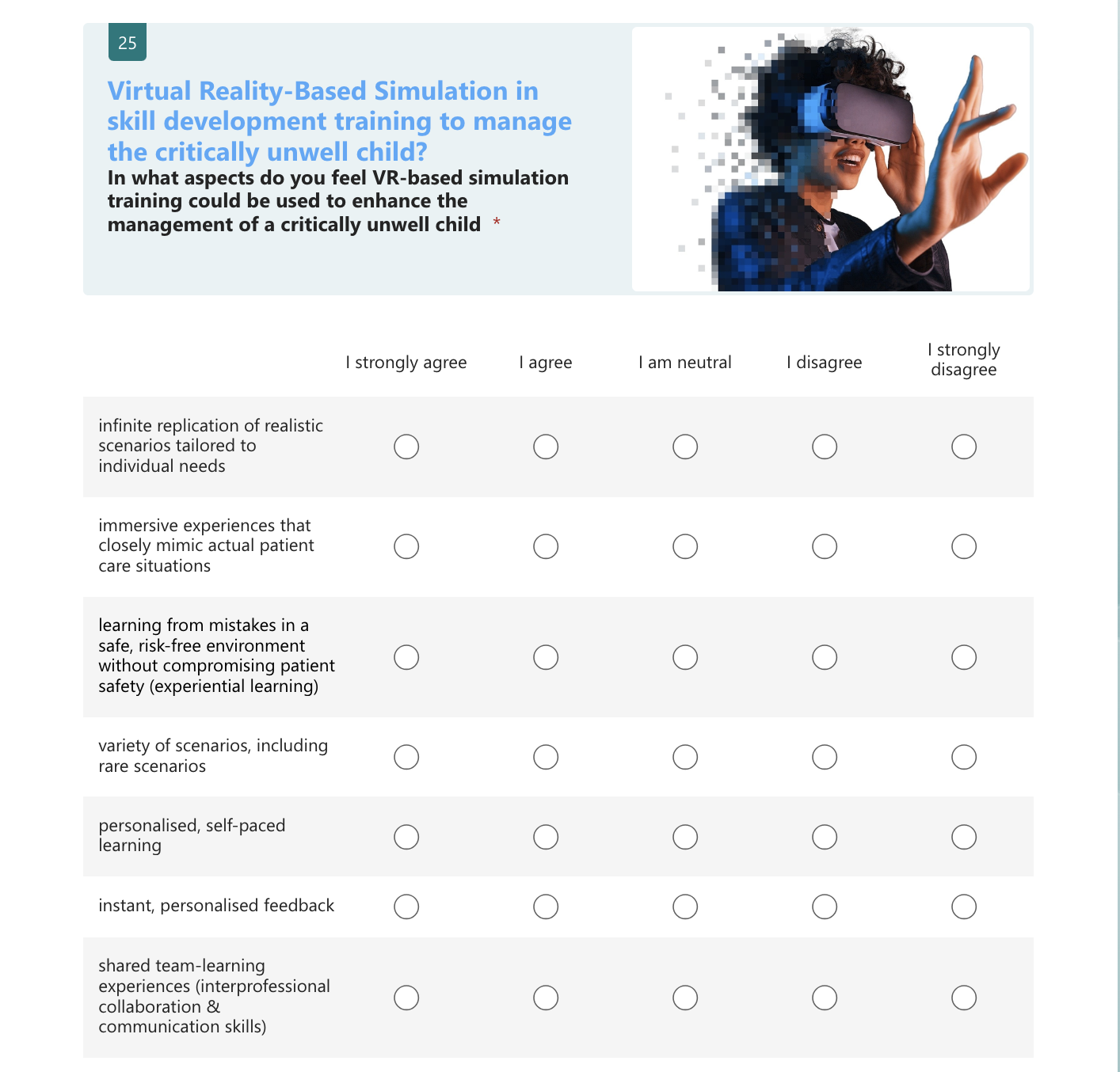


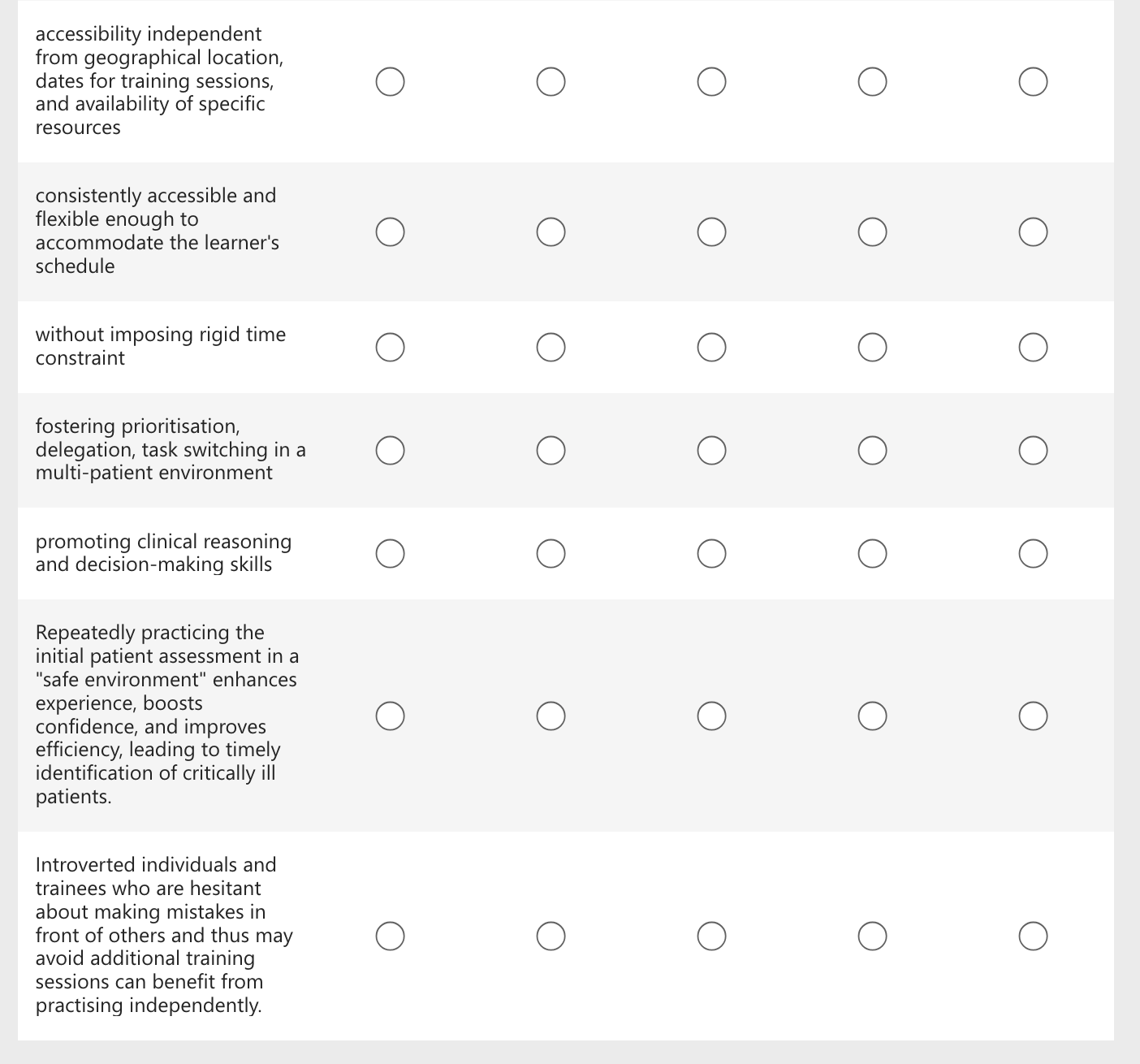
­


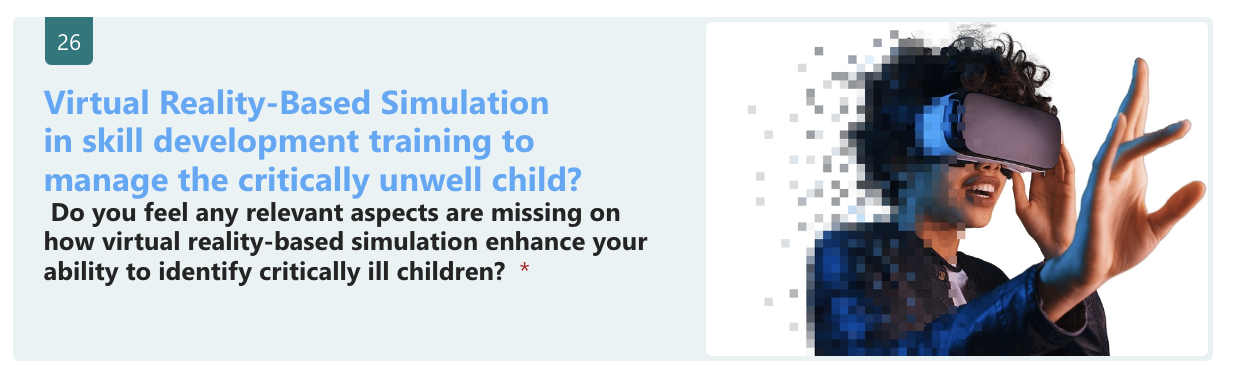

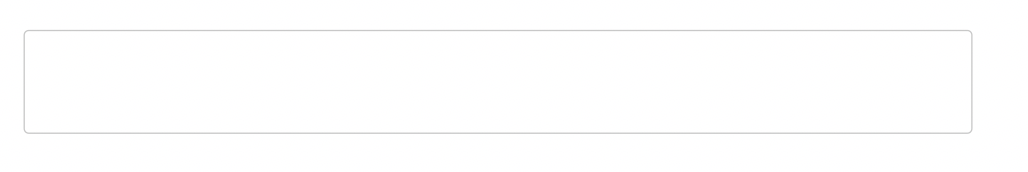


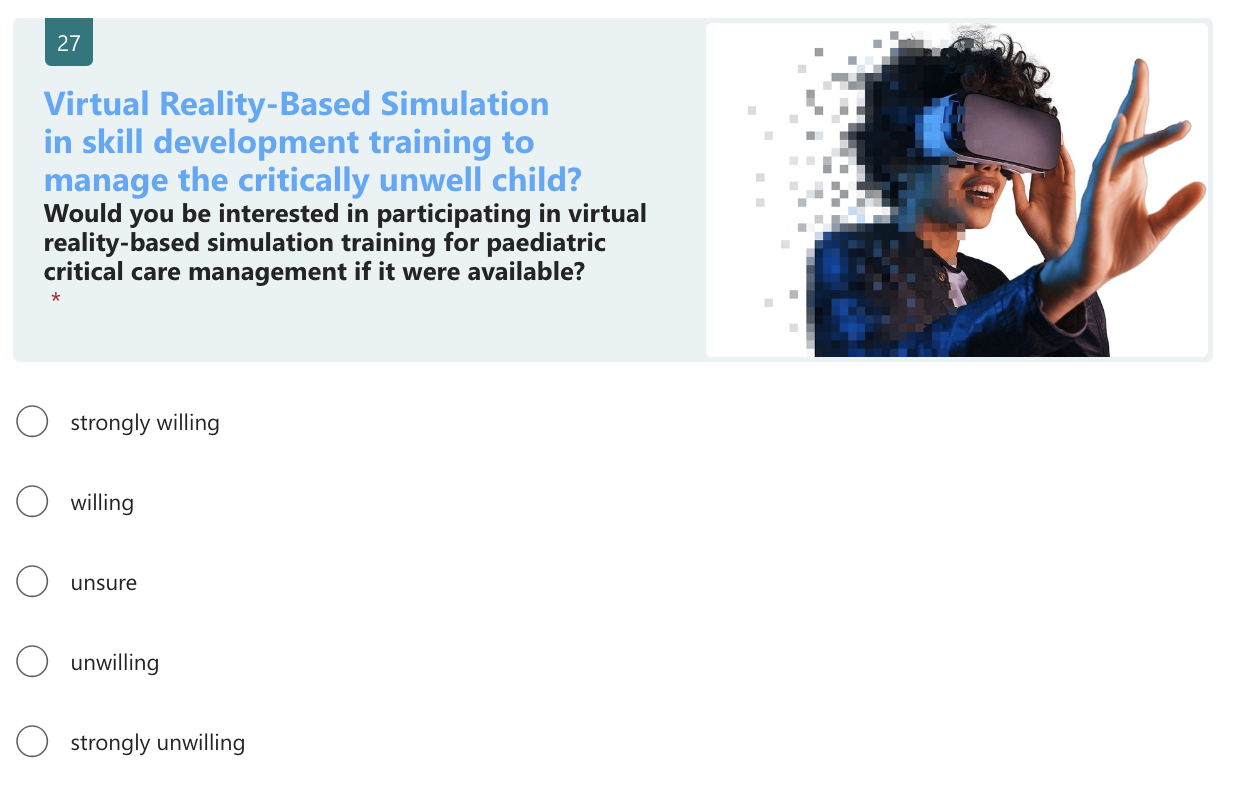


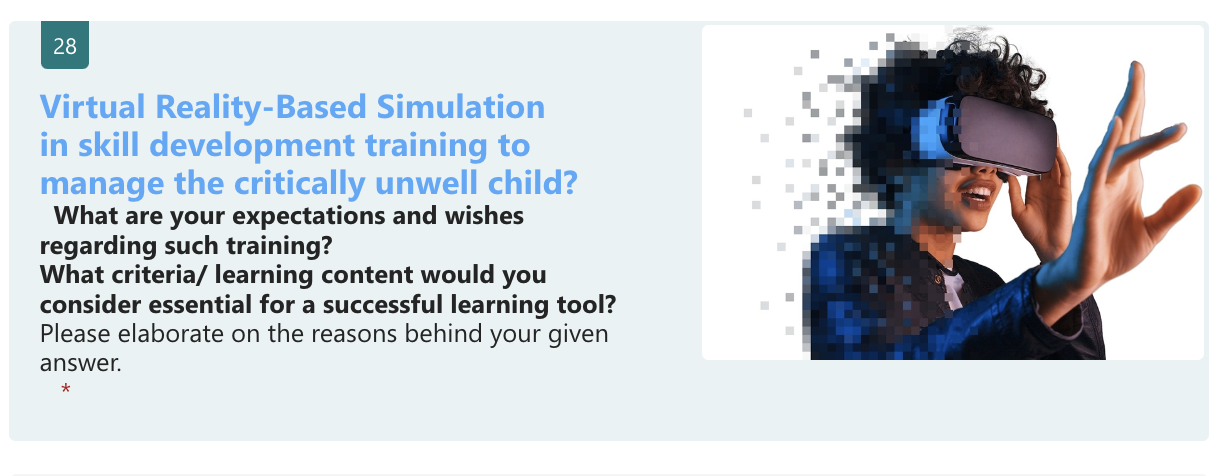

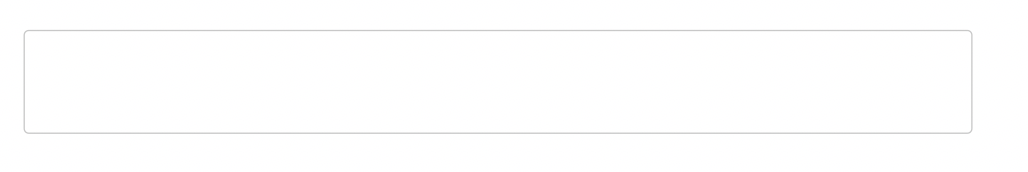


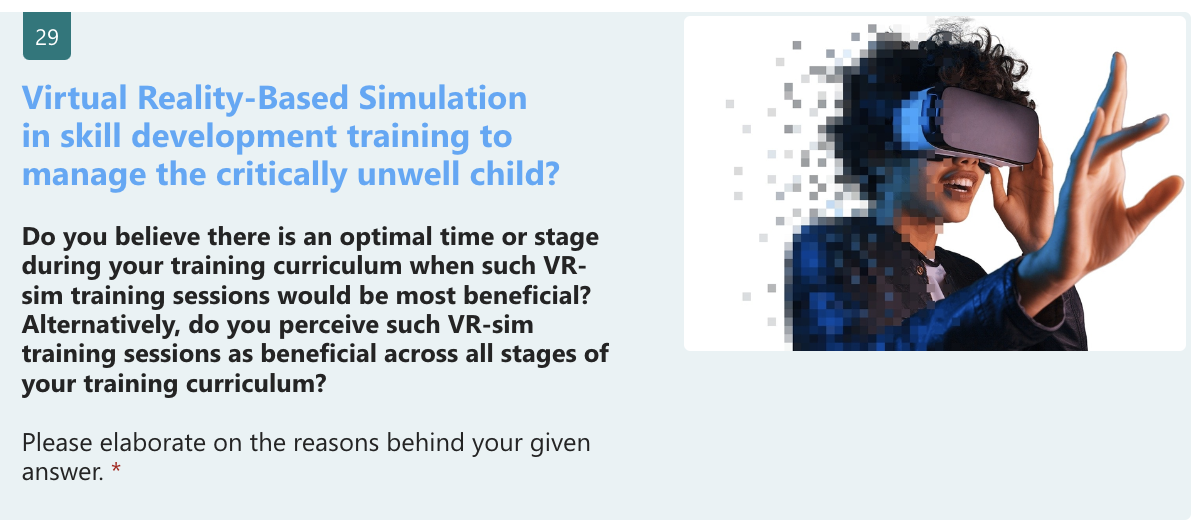

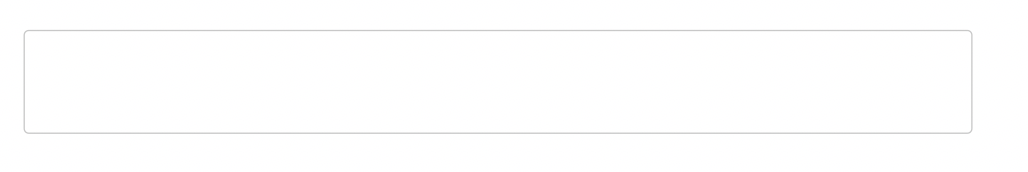


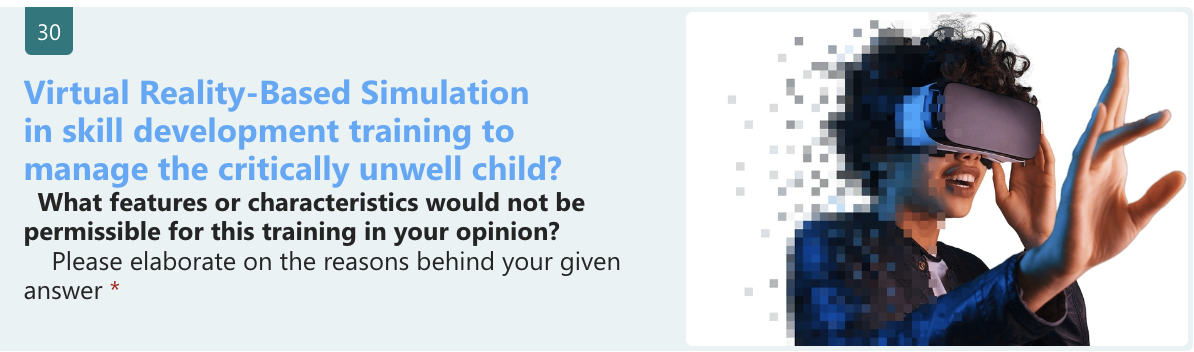

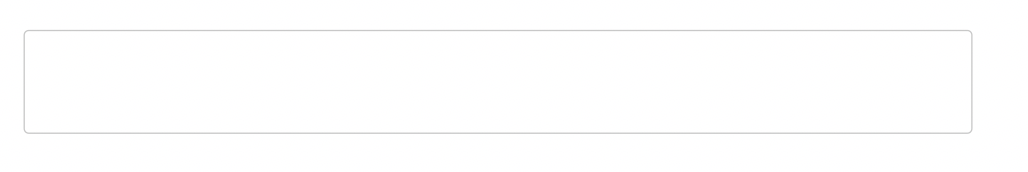


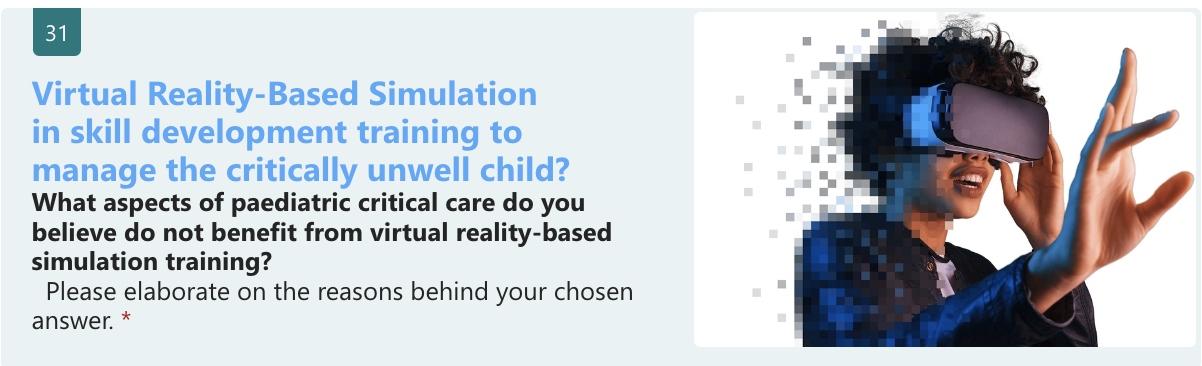

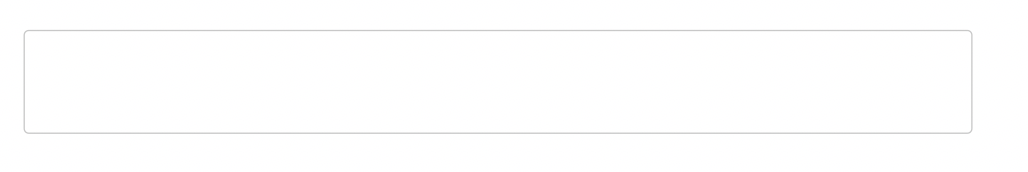


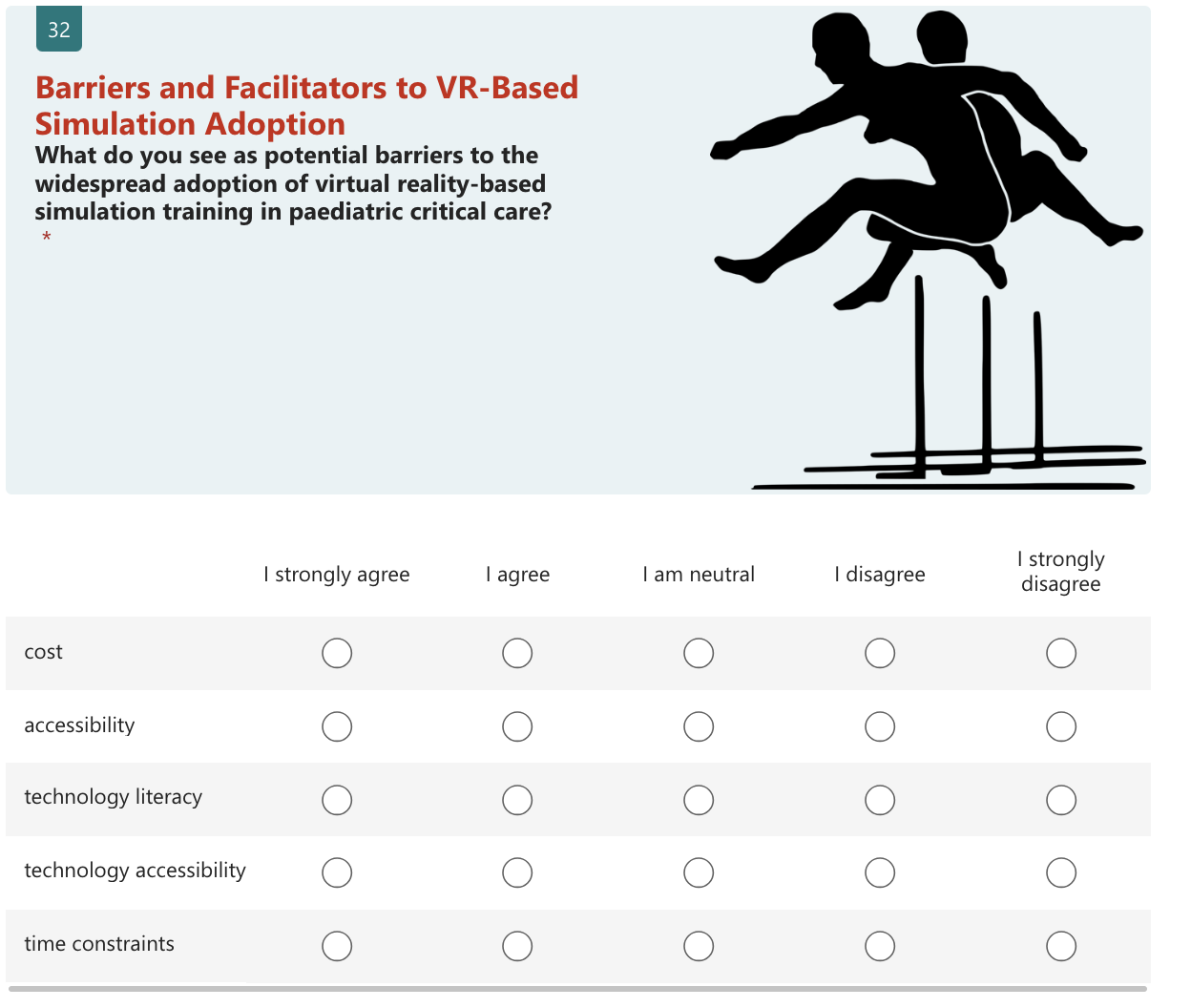


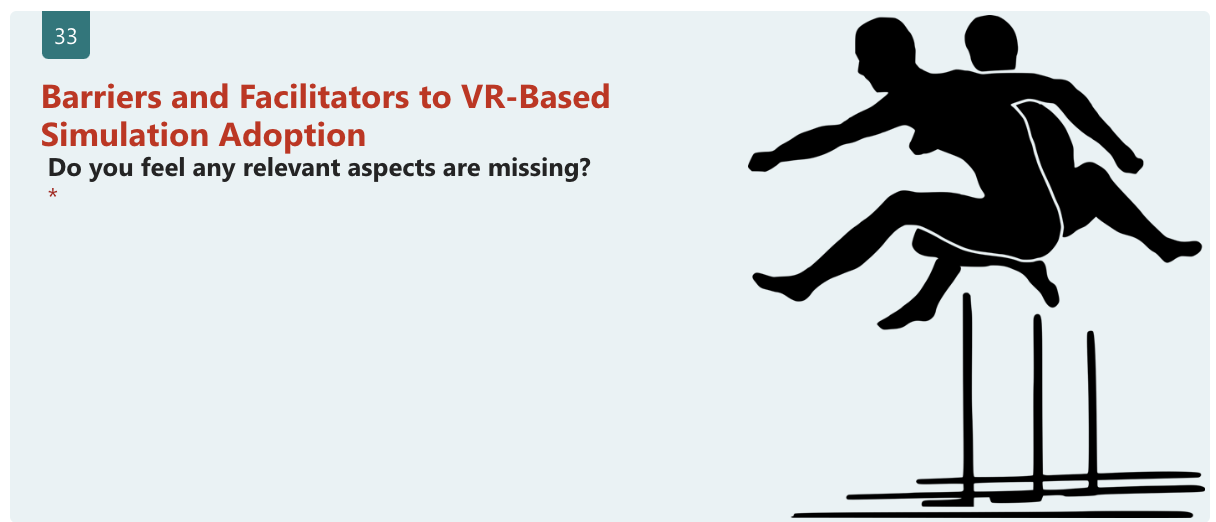

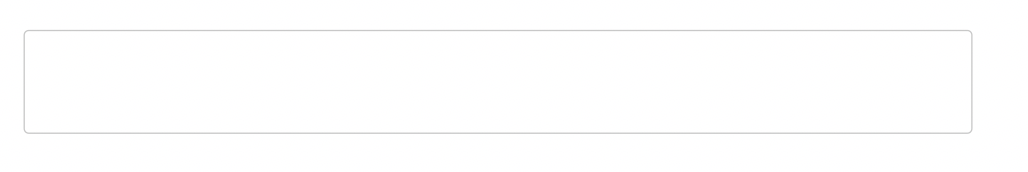


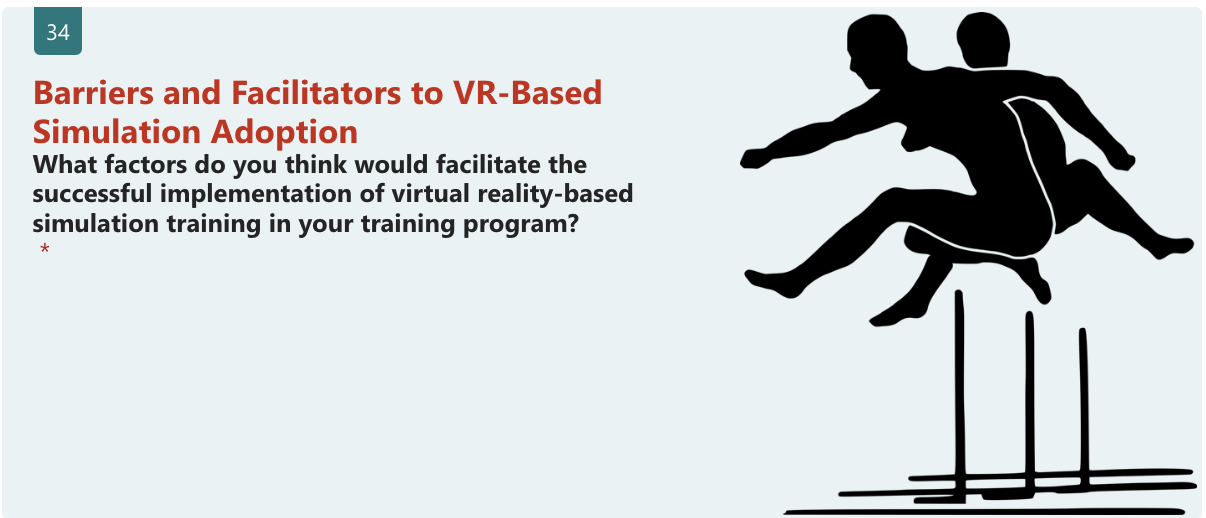

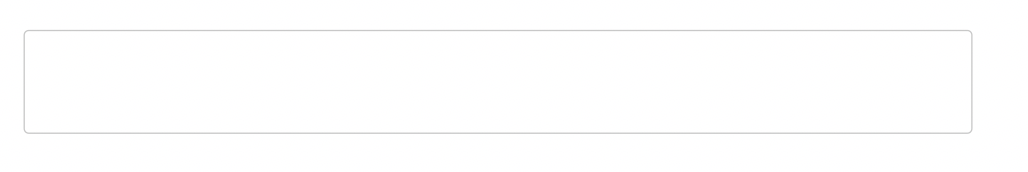


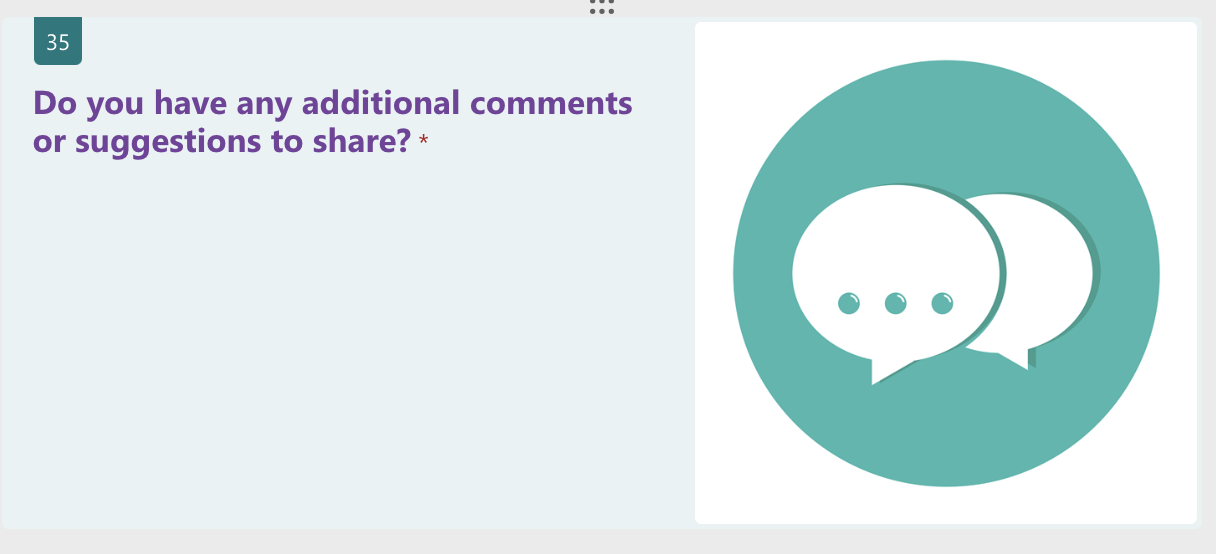

Supplement: Multimedia Appendix 2 [file formative-v10-e85743-s002.docx]
