# Supplementary material for: Virtual Reality Simulation in Postgraduate Pediatric Critical Care Training Based on Trainee Perceptions in London: Exploratory Mixed Methods Study
Source: JMIR Form Res. 2026 Jun 25;10:e85743. doi: 10.2196/85743 (PMC13296495; doi:10.2196/85743)
Supplement: Multimedia Appendix 3 [file formative-v10-e85743-s003.docx]

**Multimedia Appendix 3. Participant information sheet and consent material**

Participant Information Sheet For Paediatric Postgraduate Trainees in London

UCL Research Ethics Committee Approval ID Number: 27235.001

**YOU WILL BE GIVEN A COPY OF THIS INFORMATION SHEET**

**Title of Study:** Does integrating virtual reality-based simulation training into the current training program support paediatric trainees in cultivating the required skill set to manage a critically unwell child safely, and if so, in what manner?" Exploring paediatrics trainees' perceptions in the London deanery

**Department:** UCL Medical School (Department of Medical Education), London School of Paediatrics

**Name and Contact Details of the Researcher(s):** Dr. Anne **Claudia** Berlin (Email: anne.berlin.21@ucl.ac.uk)

**Name and Contact Details of the Principal Researcher:** Dr. Rima Chakrabarti (Email: r. chakrabarti@ucl.ac.uk) / Dr. David Harrison ([David.harrison@rcp.ac.uk](mailto:David.harrison@rcp.ac.uk))

**_________________________________________**

1. **Invitation Paragraph**

Dear colleague and fellow paediatricians,

I hope this message finds you well. I invite you to participate in an exciting medical education project exploring the role of Virtual Reality (VR) 's role in postgraduate paediatric training. This project aims to investigate how VR can be utilised to enhance the skills needed in identifying and managing an acutely unwell child.

Your valuable insights will contribute to my Master's study conducted in collaboration with UCL & RCP. Rest assured, your responses will be anonymised. The study involves completing a brief online survey, which should take no longer than twenty minutes. Additionally, you can participate in a virtual semi-structured interview lasting approx.20-30min. If you are interested in participating in the interview, then please register your interest using the link at the end of the survey. The interview will be carried out using Microsoft Teams. The conversations will be recorded.

Participation is entirely voluntary and will have no impact on your training. Your input is considered crucial to advancing our understanding of the potential applications of VR in medical training. Additional information can be found in the participation information sheet attached.

Your participation would be highly appreciated and would contribute to advancing medical education. If you have any questions or concerns, please feel free to contact me at anne.berlin.21@ucl.ac.uk.

Thank you for considering being part of this innovative study.

Best wishes

Claudia Berlin

1. **What is the project’s purpose?**

This study is designed to explore your perspective on the potential educational advantages of virtual reality-based simulation training for developing and enhancing skills in identifying and managing critically ill children. VR simulation seems to provide infinitely repeatable scenarios, delivering immersive and standardised simulations with immediate, personalised feedback. This approach's flexible and engaging nature is supposed to enable learners to assess, diagnose, and treat virtual patients as they would in real life, offering limitless opportunities for improvement. The aim of this study is to explore how it can be used to optimise how it can be used to your learning experience enhancing skill sets to identify critically - ill children.

1. **Why have I been chosen?**

All paediatric trainees and foundation doctors with an interest in paediatrics are eligible to participate, as I am interested in understanding trainees' perceptions of the role of VR in developing their skills in managing an unwell child.

1. **Do I have to take part?**

Study participation is entirely voluntary. And there will be no impact to your training if you choose to not participate.

1. **What will happen to me if I take part?**

If you opt to participate, you will be asked to keep this information sheet. By starting to answer the questionnaire you are indicating your consent to participate in the online survey.

If you are interested in participating in a semi-structured interview (lasting approx. 20-30min) then please follow the link in the message to register your interest and date you are free. Once this is done, you will be sent a consent form that must be completed prior to the discussion. An email confirming your participation will be sent in due course too.

1. **Will I be recorded and how will the recorded media be used?**

Interviews will be carried out remotely using Microsoft Teams. They will be recorded to enable data analysis and securely stored temporarily. Transcriptions will be generated, and the recordings will be subsequently discarded following the completion of the MSc. Transcripts will be kept and safely stored until 1^st^ July 2025 in case of potential publication of study findings.

1. **What are the possible disadvantages and risks of taking part?**

There are no expected disadvantages or risks associated with your participation.

However, while it is not anticipated that any sensitive or difficult information will be disclosed, drawing on personal experience might potentially be distressing. Therefore, for mental health and well-being support, I would like to highlight the support services provided by the NHS medical line (phone:111), or by the Samaritans (phone 166123). These services can be contacted 24/7 for listening service or directing to further adequate support.

To minimise this risk, survey questions can be omitted. Regarding the interview a basic question guide will also be made available to ensure you are aware of the nature of the questions being asked. Furthermore, all participants can leave the interview at any during their conduction without indicating any reasons. Furthermore, you can withdraw your consent **within four weeks after interview completion.**

1. **What are the possible benefits of taking part?**

Participation in this study will not yield any direct personal benefits. Nevertheless, the study's intention is to utilise the findings to shape the role of Virtual Reality (VR) in paediatric training. The insights gathered may contribute to the enhancement of future training programs through the integration of VR-based simulation sessions, refining the current training landscape. Your insights will be valuable in aiding medical educators and training directors to comprehend the potential advantages and obstacles associated with adopting VR-sim-based technology within the scope of innovative and effective training methodologies aiming to refine paediatrics training.

1. **What if something goes wrong?**

In case you wish to raise a complaint please contact Dr. R. Chakrabarti (principal investigator) or myself. Contacts are stated above. In case you feel your complaint has not been handled to your satisfaction you can contact the Chair of the UCL Research Ethics Committee via: [ethics@ucl.ac.uk](mailto:ethics@ucl.ac.uk).

1. **Will my taking part in this project be kept confidential?**

All information gathered will be kept strictly confidential. All gathered data will be anonymised and securely stored on UCL's Data Safe Haven, a portal to store and handle data securely.

All responses will be anonymised and be kept confidential. In the event of information being disclosed that may affect patient safety, then confidentiality may be breached.

The collection of unnecessary personal data will be minimised in this study. However, if you opt to participate in the interview, your name will be obtained through online consent form. To ensure confidentiality, each participant will be assigned a study ID code before interview participation. Furthermore, your contribution will be recorded. However, recordings will be destroyed after transcription.

1. **What will happen to the results of the research project?**

The results obtained will mainly be used for my MSc thesis in the context of the postgraduate medical education (MedEd) course. In addition, future findings might be used for further research and communicated to a broader MedED community. After finishing the research project, all obtained observations will be made available.

1. **Who has reviewed the study?**

As part of the Master’s study, this study has been approved by UCL Ethics Committee.

1. **Contact for further information**

Anne Claudia Berlin

Email: anne.berlin.21@ ucl.ac.uk

If you are interested in participating in the INTERVIEW, then please register your interest using the link at the end of the survey.

**Thank you for reading this information sheet and for considering to take part in this research study.**


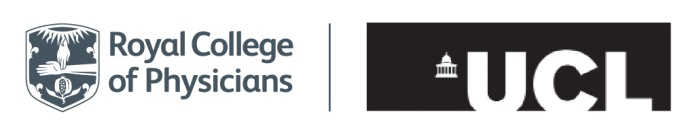


**CONSENT FORM FOR PAEDIATRIC POSTGRADUATE MEDICAL TRAINEES IN RESEARCH STUDIES**

**Please complete this form after you have read the Information Sheet.**

Participant identification number: P0X

**Title of Study:** Does integrating virtual reality-based simulation training into the current training program support paediatric trainees in cultivating the required skill set to manage a critically unwell child safely, and if so, in what manner?" Exploring paediatrics trainees' perceptions in the London deanery

**Department:** UCL Medical School (Department of Medical Education), London School of Paediatrics

**Name and Contact Details of the Researcher(s):** Dr. Anne Claudia Berlin (Email: anne.berlin.21@ucl.ac.uk)

**Name and Contact Details of the Principal Researcher:** Dr. Rima Chakrabarti (Email: r. [chakrabarti@ucl.ac.uk](mailto:chakrabarti@ucl.ac.uk)) / Dr. David Harrison ([David.harrison@rcp.ac.uk](mailto:David.harrison@rcp.ac.uk))

**Name and Contact Details of the UCL Data Protection Officer:** Alexandra Potts (Email: data- protection@ ucl.ac.uk)

**This study has been approved by the UCL Research Ethics Committee: Project ID number:** 27235.001 Thank you for considering taking part in this research. The person organising the research must explain the project to you before you agree to take part. If you have any questions arising from the Information Sheet or explanation already given to you, please ask the researcher before you decide whether to join in. You will be given a copy of this Consent Form to keep and refer to at any time.

**I confirm that I understand that by ticking/initialling each box below I am consenting to this element of the study. I understand that it will be assumed that unticked/initialled boxes means that I DO NOT consent to that part of the study. I understand that by not giving consent for any one element that I may be deemed ineligible for the study.**

***If you agree, please tick the initial box:* ↓**

| 1. Hereby, I confirm that I have read and understood the information sheet for the study above. I confirm that I have had the opportunity to consider the information and what will be expected of me. I have also had the opportunity to ask questions and have had these answered satisfactorily. | O_YES  O_NO |
| --- | --- |
| 2.Hereby, I confirm that I would like to take part in the proposed online semi-structured interview. I also confirm that I have no objections to the conversation held on Microsoft Teams being recorded. | O_YES  O_NO |
| 3. I understand that I am free to withdraw my hereby given consent **within four weeks after the interview completion** without giving any reason or my legal rights being affected. I understand that if I decide to withdraw, any data I have provided up to that point will be destroyed unless I agree otherwise. My respective contributions will not be transcribed or included in the data analysis. Having received an anonymised study code before participating will enable the precise deletion of my individual data inputs. | O_YES  O_NO |
| 4.I consent to the processing of my personal data for the purpose explained to me. I understand that such information will be handled in accordance with all applicable data protection legislation (public task will be the lawful basis for processing). | O_YES  O_NO |
| 5. I understand that relevant sections of my given data during this study may be reviewed by Dr. Berlin & Dr. Chakrabarti the principal investigator, and responsible individuals from the University, where it is relevant to my taking part in this research, monitoring purposes and audit purposes. | O_YES  O_NO |
| 6.I understand that all personal information will remain confidential, and all efforts taken that I cannot be identified. All gathered data (written, recordings) will be transcribed, remain anonymised and temporarily stored at a safe place. It will not be possible to identify me in the thesis-write-up, presentations or any potential future publication.  I understand that the collection of unnecessary personal data will be minimised in this study. However, I am aware that participants' names will be obtained through online consent forms. I understand that before my /our engagement in the study, and each participant will be assigned a study ID code to ensure confidentiality. | O_YES  O_NO |
| 7. I understand that in case of a disclosed risk to patient or staff safety during the discussion, the TPD will need to be informed. | O_YES  O_NO |
| 8. I understand that the information I have submitted will be written up and potentially published. | O_YES  O_NO |
| 9. I understand the potential risks of participating in this study and that support will be available to me should I become distressed during the course of this study.  I understand that while it is not anticipated that any sensitive or difficult information will be disclosed, drawing on personal experience might potentially be distressing. I understand that support services, the NHS medical line (phone:111), or the Samaritans (phone 166123) can be 24/7 be contacted for listening service if required. To minimise this risk, I understand that a basic question guide will also be made available to ensure that I am aware of the nature of the questions being asked. | O_YES  O_NO |
| 10. I understand that the data obtained will not be made available to any commercial organisations but is solely the responsibility of the researchers undertaking this study. | O_YES  O_NO |
| 11. I agree to provide data as part of my involvement in this study and I understand I will not gain any direct personal or financial benefit from them now or in the future. | O_YES  O_NO |
| 12. I agree to the use of anonymised quotes in research reports and publications. | O_YES  O_NO |
| 13. I am aware of whom I should contact if I wish to file a complaint. | O_YES  O_NO |
| 14. I voluntarily take part in this study. | O_YES  O_NO |

**If you would like your contact details to be retained so that you can be contacted in the future by UCL researchers who would like to invite you to participate in follow up studies to this project, or in future studies of a similar nature, please tick the appropriate box below.**

|  | Yes, I would be happy to be contacted in this way |  |
| --- | --- | --- |
|  | No, I would not like to be contacted |  |

_________________________ ________________ ___________________

Name of participant Date Signature

_________________________ ________________ ___________________

Researcher Date Signature
