# Supplementary material for: Virtual Reality Simulation in Postgraduate Pediatric Critical Care Training Based on Trainee Perceptions in London: Exploratory Mixed Methods Study
Source: JMIR Form Res. 2026 Jun 25;10:e85743. doi: 10.2196/85743 (PMC13296495; doi:10.2196/85743)
Supplement: Multimedia Appendix 4 [file formative-v10-e85743-s004.docx]

**Multimedia Appendix 4. Semi-structured interview guide**

Introduction:

- Could you briefly introduce yourselves, stating your current level of paediatrics training, whether

you had any training experience in (paediatric) emergency medicine or (paediatric) intensive care and whether you have ever cared for a critically ill child?

Understanding current practices: Perceptions of current training methods and training needs:

- What skills do you feel are required to manage a critically ill child and which aspects do you find most challenging to require in your current training? *(prompts: Can you describe/explain further? What do you mean specifically?)*
- Have you ever attended specific training for managing critically ill children? And if yes, was this helpful and in what way? *(prompt: Could you explain further?)*

Initial impressions of virtual reality (VR)-based simulation training in paediatrics

- Have you ever experienced VR-based sim technology in medical education training, in particular in paediatrics? If yes, how did these experiences impact your learning and clinical preparedness in general and in comparison, to traditional methods? *(prompts: Could you explain further? What do you specifically mean?)*
- In what ways do you think VR-sim training might complement your current clinical training in paediatrics? *(prompt: Can you describe further?)*

**Future directions/VR-sim training integration into current training curricula:**

- - What support, resources and strategies do you think would be necessary to successfully facilitate the widespread adoption and smooth implementation of VR-simulation training in paediatric education? *(prompts: What do you specifically mean by that? Could you explain further?)*
  - Which other fields of paediatric training do you envision to benefit from VR-sim training? *(prompts: What do you specifically mean by that? Could you explain further?)*

**Challenges and Concerns:**

- - How feasible do you think it is to integrate VR-sim training into the current paediatric training curriculum and where do you identify /expect challenges and barriers in the implementation of VR-sim training? *(prompts: What do you specifically mean by that? Could you explain further?)*

Further comments?
