# Supplementary material for: Virtual Reality Simulation in Postgraduate Pediatric Critical Care Training Based on Trainee Perceptions in London: Exploratory Mixed Methods Study
Source: JMIR Form Res. 2026 Jun 25;10:e85743. doi: 10.2196/85743 (PMC13296495; doi:10.2196/85743)
Supplement: Multimedia Appendix 5 [file formative-v10-e85743-s005.docx]

**Multimedia Appendix 6. Detailed Psychometric Evaluation of Survey Instrument**

Corrected item–total correlations ranged from –0.33 to 0.78, with the majority of items exceeding the commonly recommended threshold of 0.30, indicating generally satisfactory item performance. A small subset of items demonstrated lower or negative item–total correlations; these were retained due to their conceptual relevance and because “alpha if item deleted” analyses did not demonstrate meaningful improvement in overall internal consistency. Such findings likely reflect the conceptual breadth of the instrument and heterogeneity typical of exploratory educational perception measures rather than clear item malfunction.

Item-level response distributions demonstrated ceiling effects for items assessing perceived educational usefulness and realism, with over 60% (18/30) of respondents selecting “agree” or “strongly agree” for several attitudinal items. In contrast, some barrier-related items exhibited floor-pattern clustering at the lower end of the response scale. These distributional patterns are consistent with perception-based educational evaluations and may have reduced discriminative capacity for some attitudinal domains.
