# Supplementary material for: Virtual Reality Simulation in Postgraduate Pediatric Critical Care Training Based on Trainee Perceptions in London: Exploratory Mixed Methods Study
Source: JMIR Form Res. 2026 Jun 25;10:e85743. doi: 10.2196/85743 (PMC13296495; doi:10.2196/85743)
Supplement: Multimedia Appendix 6 [file formative-v10-e85743-s006.docx]

**Multimedia Appendix 7. Participant characteristics**

| Characteristics | Total= 30, N (%) |  |
| --- | --- | --- |
| Gender |  |  |
| Male | 13/30 (43 %) |  |
| Female | 16/30 (53 %) |  |
| Non-binary | 1/30 (3 %) |  |
| Age range |  |  |
| 23-25 years | 2/30 (7 %) |  |
| 26-29 years | 6/30 (20 %) |  |
| 30-33 years | 9/30 (30 %) |  |
| 34-37 years | 9/30 (30 %) |  |
| 38-40 years | 3/30 (10 %) |  |
| 41-45 years | 1/30 (3 %) |  |
| Years of clinical experience |  |  |
| 0,5 | 2/30 (7 %) |  |
| 1 | 3/30 (10 %) |  |
| 2 | 3/30 (10 %) |  |
| 3 | 4/30 (13 %) |  |
| 4 | 2/30 (7 %) |  |
| 5 | 3/30 (10 %) |  |
| 6 | 5/30 (17 %) |  |
| 7 | 6/30 (20 %) |  |
| 8 | 2/30 (7 %) |  |
| Previous paediatric intensive care or emergency medicine rotation |  |  |
| Yes | 24/30 (80%) |  |
| No | 6/30 (20%) |  |
| Paediatric subspecialty/alternative specialty* aspiration |  |  |
| General paediatrics | 5/30 (17 %) |  |
| Intensive care | 2/30 (7 %) |  |
| Emergency medicine | 2/30 (7 %) |  |
| Neonatology | 2/30 (7 %) |  |
| Medical education* | 1/30 (3 %) |  |
| Clinical genetics* | 2/30 (7 %) |  |
| Oncology | 2/30 (7 %) |  |
| Diabetes and Endocrinology | 1/30 (3 %) |  |
| Infectious diseases | 2/30 (7 %) |  |
| Cardiology | 2/30 (7 %) |  |
| Inherited metabolic medicine | 1/30 (3 %) |  |
| Community paediatrics | 3/30 (10 %) |  |
| Nephrology | 1/30 (3 %) |  |
| Neurology | 1/30 (3 %) |  |
| Respiratory medicine | 1/30 (3 %) |  |
| Gastroenterology | 1/30 (3 %) |  |
| INTERVIEW |  |  |
| Interviewee 1 (I*1*) | 35 years, female, ST 6, general paediatrics, ICU experienced | Interview length: 75 min |
| Interviewee 2 (I*2*) | 38 years, male, ST 8, general paediatrics with neonatology, ICU experienced | Interview length: 91 min |
