# Supplementary material for: Virtual Reality Simulation in Postgraduate Pediatric Critical Care Training Based on Trainee Perceptions in London: Exploratory Mixed Methods Study
Source: JMIR Form Res. 2026 Jun 25;10:e85743. doi: 10.2196/85743 (PMC13296495; doi:10.2196/85743)
Supplement: Multimedia Appendix 7 [file formative-v10-e85743-s007.docx]

**Multimedia Appendix 8. Trainee perceptions of current approaches to skill development for recognising and managing critically ill children.**


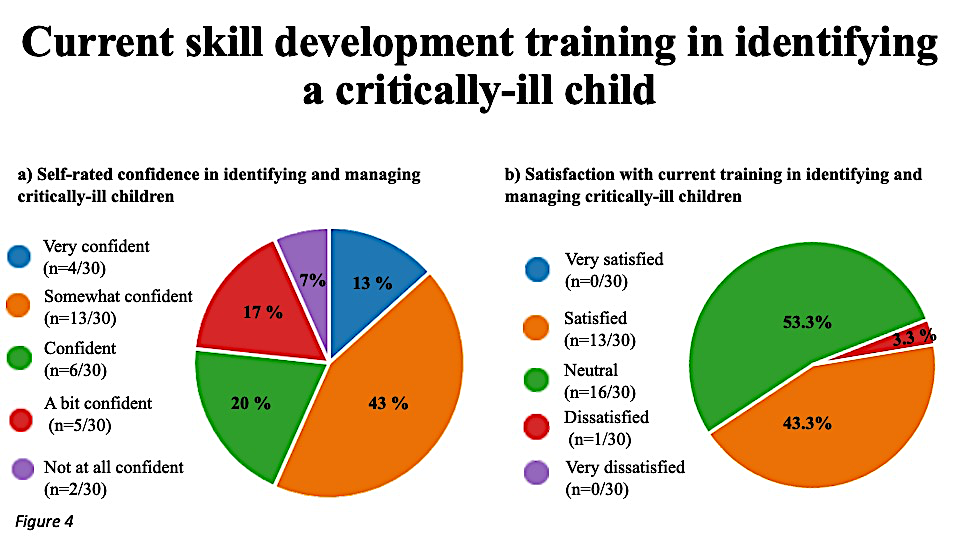


Multimedia Appendix 8: Current skill- development-training in identifying a critically ill child.

**Panel A)** *Self-rated confidence of LSP trainees (n=30) in identifying and managing critically ill children through their current training. Most participants rated themselves as "somewhat confident" or "confident," indicating overall moderate confidence levels.*

**Panel B)** *LSP trainees' satisfaction with their current training in recognizing critically ill children (n=30), measured on a five-point Likert scale. Most responses were "neutral" or "satisfied," reflecting moderate overall satisfaction. Notably, no participants selected "very satisfied" or "very dissatisfied."*
