# Supplementary material for: Virtual Reality Simulation in Postgraduate Pediatric Critical Care Training Based on Trainee Perceptions in London: Exploratory Mixed Methods Study
Source: JMIR Form Res. 2026 Jun 25;10:e85743. doi: 10.2196/85743 (PMC13296495; doi:10.2196/85743)
Supplement: Multimedia Appendix 8 [file formative-v10-e85743-s008.docx]

**Multimedia Appendix 9. Knowledge and familiarity with artificial intelligence and virtual reality among LSP trainees**

*
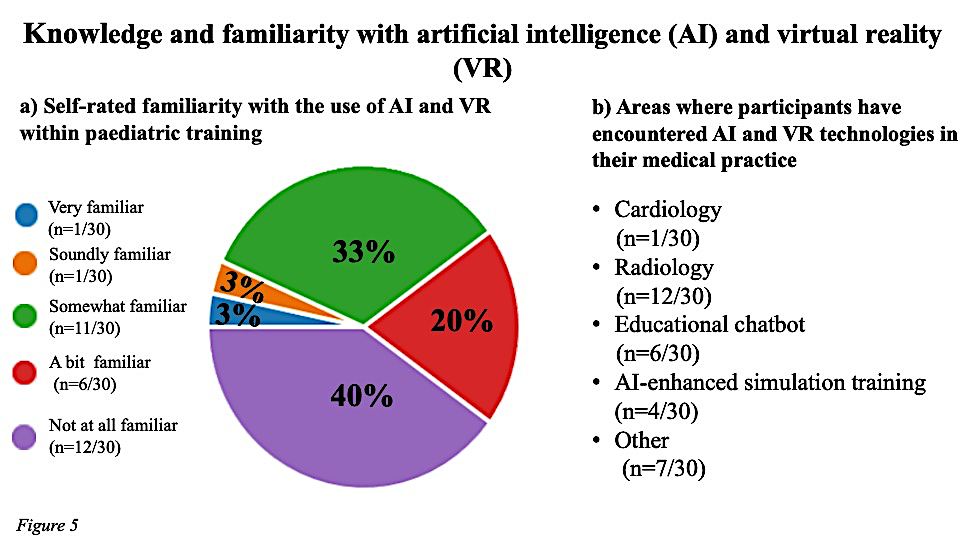
*

**Multimedia Appendix 9. Knowledge and familiarity with artificial intelligence and virtual reality among LSP trainees (n=30).
Panel A):** Participants’ self-assessed familiarity with artificial intelligence (AI) and virtual reality (VR) in paediatric training.
**Panel B):** Clinical contexts in which participants had encountered AI and VR technologies in medical practice.
