# Supplementary material for: Virtual Reality Simulation in Postgraduate Pediatric Critical Care Training Based on Trainee Perceptions in London: Exploratory Mixed Methods Study
Source: JMIR Form Res. 2026 Jun 25;10:e85743. doi: 10.2196/85743 (PMC13296495; doi:10.2196/85743)
Supplement: Multimedia Appendix 9 [file formative-v10-e85743-s009.docx]

**Multimedia Appendix 10. Detailed attitudinal response distributions regarding trainees’ perceptions of the educational role of VR-simulation in paediatric critical care training.**


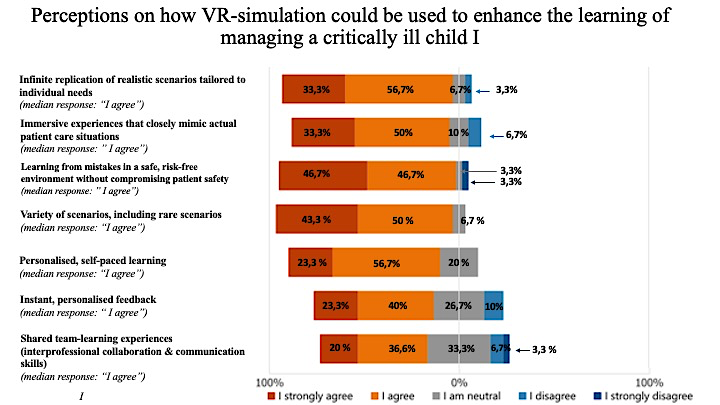


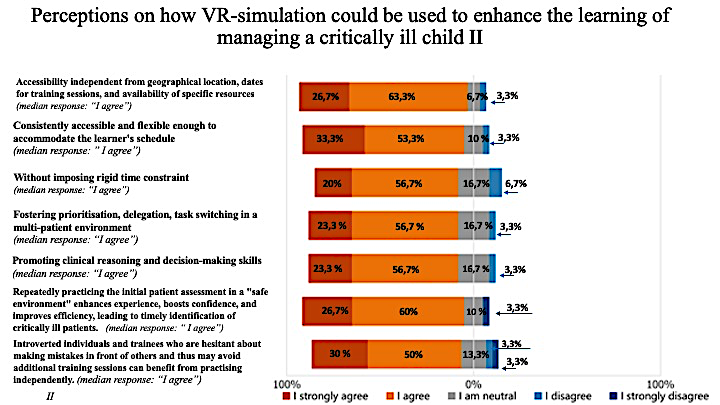


Multimedia Appendix 10. Detailed attitudinal response distributions regarding trainees’ perceptions of the educational role of VR-simulation in paediatric critical care training.
