# Supplementary material for: Virtual Reality Simulation in Postgraduate Pediatric Critical Care Training Based on Trainee Perceptions in London: Exploratory Mixed Methods Study
Source: JMIR Form Res. 2026 Jun 25;10:e85743. doi: 10.2196/85743 (PMC13296495; doi:10.2196/85743)
Supplement: Multimedia Appendix 10 [file formative-v10-e85743-s010.docx]

**Multimedia Appendix 11. Perceived barriers and facilitators to the use and implementation of VR-simulation for paediatric critical care skills development.**

| **Barriers** | **N (%)** |
| --- | --- |
| Cost (e.g., hardware/software, training faculty) | 24/30 (80%) |
| Lack of familiarity and knowledge among all stakeholders | 5/30 (17%) |
| Lack of organisational or/and senior management/leadership support | 3/30 (10 %) |
| Lack of trained facilitators | 4/30 (13%) |
| Trainer / facilitator reluctance and resistance | 2/30 (7%) |
| Lack of technological literacy | 17/30 (57%) |
| Lack of technology availability and accessibility (software, hardware, stable WI-FI) | 15/30 (50%) |
| Accessibility (“unlikely to be accessible to poorer areas/countries”, so if more researchers focus on technology in medical education that is not widely accessible | 17/30 (57%) |
| Reluctance to change | 1/30 (3%) |
| Adverse side-effects (e.g., “motion-sickness”, “dizziness”, “challenge for people with glasses) | 1/30 (3%) |
| Lack of protected teaching time (e.g., “clinical workload”, “understaffing”) | 1/30 (3%) |
| “Unsure due to lack of experience | 6/30 (20%) |
| **Supporting Factors/ Facilitators** | **N (%)** |
| Organisational and senior leadership/management support and endorsement (incl. NHS, HEE) | 10/30 (33%) |
| Sufficient funding | 7/30 (23%) |
| Training courses for clinical staff (trainers and trainees) | 10/30 (33%) |
| Events to increase familiarity and knowledge (e.g., taster days at conferences, training days; general, instructional information on VR-simulation as an educational tool and guidelines on how to use VR, e.g., YouTube-videos or brochures) | 15/30 (50%) |
| Research substantiating its educational benefit and value | 5/30 (17%) |
| VR-advocates/trailblazers among all stakeholder subgroups (locally and generally) | 8/30 (27%) |
| Access to sufficient equipment, user-friendly hard- and software, which is easy to maintain and store | 6/30 (20%) |
| Access from everywhere at anytime | 1/30 (3%) |
| Dedicated time allocated for training | 5/30 (17%) |
| Implementation employing a structure within local training programmes | 3/30 (10%) |
| Broad scope of high-fidelity, realistic scenarios enabling practice of situations you cannot practice well employing a mannequin (ideally work-place adapted) | 3/30 (10%) |

*“N” refers to the number of participants (out of n=30) who indicated a respective aspect*
