# Supplementary material for: Virtual Reality Simulation in Postgraduate Pediatric Critical Care Training Based on Trainee Perceptions in London: Exploratory Mixed Methods Study
Source: JMIR Form Res. 2026 Jun 25;10:e85743. doi: 10.2196/85743 (PMC13296495; doi:10.2196/85743)
Supplement: Checklist 1 [file formative-v10-e85743-s011.docx]

**Multimedia Appendix 5. CHERRIES checklist for reporting of internet e-surveys**

Checklist for Reporting Results of Internet E-Surveys (CHERRIES) tailored to the study: “Virtual Reality Simulation for Postgraduate Paediatric Critical Care Training: An Exploratory Mixed Methods Study of Trainee Perceptions in London”

| Item Category | Checklist Item | Reported in This Study | Location in Manuscript |
| --- | --- | --- | --- |
| Design | Describe survey design | This was a cross-sectional online survey embedded within an exploratory mixed methods study. The target population was paediatric trainees in the London School of Paediatrics (approximately 450 trainees across ST1–ST8). The sample was a non-probability convenience sample recruited through official WhatsApp groups. | Methods – Study Design and Research Approach; Setting, Participants, and Recruitment |
| IRB approval and informed consent | IRB approval | Ethical approval was obtained through the appropriate institutional process at University College London as part of the MSc in Medical Education. | Methods – Ethical Considerations |
| IRB approval and informed consent | Informed consent | Potential participants received a participant information sheet before accessing the survey. Completion of the questionnaire constituted implied consent. Interview participants provided written informed consent. The information sheet stated the study purpose, investigator identity, voluntary nature of participation, and data handling arrangements. | Methods – Setting, Participants, and Recruitment; Ethical Considerations; Multimedia Appendix 4 |
| IRB approval and informed consent | Data protection | Survey responses were collected anonymously, and no directly identifiable personal information was retained in the analytical dataset. Data were stored securely in accordance with institutional requirements. | Methods – Ethical Considerations |
| Development and pre-testing | Development and testing | A 35-item questionnaire was developed following a focused literature review and consultation with paediatric trainees, simulation educators, and a paediatric critical care lead. The questionnaire and interview guide underwent expert review for clarity, conceptual coverage, and theoretical alignment. Piloting with 3 paediatric trainees assessed clarity, usability, cognitive load, approximate completion time, and technical functionality before fielding. | Methods – Instrument Development and Theoretical Foundation; Expert Review and Piloting |
| Recruitment process | Open survey versus closed survey | This was a closed survey distributed only to the relevant trainee population via official London School of Paediatrics WhatsApp groups. | Methods – Setting, Participants, and Recruitment |
| Recruitment process | Contact mode | Initial contact with potential participants was made online through WhatsApp group messages containing the participant information sheet and survey link. | Methods – Setting, Participants, and Recruitment |
| Recruitment process | Advertising the survey | The survey was announced through the London School of Paediatrics WhatsApp groups. The invitation included a brief description of the study, participant information sheet, survey link, and optional interview registration link. Invitations were re-issued periodically during data collection to mitigate low participation. | Methods – Setting, Participants, and Recruitment; Multimedia Appendix 4 |
| Survey administration | Web/E-mail | The survey was a web-based questionnaire hosted on Microsoft Forms. Responses were captured automatically through the platform and exported for analysis; no manual data entry from paper or email responses was required. | Methods – Survey Instrument and Domains |
| Survey administration | Context | The survey link was distributed in official trainee WhatsApp groups used by the London School of Paediatrics for postgraduate communication. These groups are routinely used by paediatric trainees for deanery-related updates and therefore provided direct access to the intended sample, although they may have favoured trainees who were more engaged with digital communications. | Methods – Setting, Participants, and Recruitment; Strengths and Limitations |
| Survey administration | Mandatory/voluntary | Participation in the survey was entirely voluntary. | Methods – Setting, Participants, and Recruitment |
| Survey administration | Incentives | No financial or non-financial incentives were offered. | Methods – Setting, Participants, and Recruitment |
| Survey administration | Time/Date | Data were collected between April and July 2024. | Methods – Setting, Participants, and Recruitment |
| Survey administration | Randomization of items or questionnaires | Questionnaire items were not randomized. All participants received the same item order. | Methods – Survey Instrument and Domains |
| Survey administration | Adaptive questioning | Adaptive questioning was not used. All participants were presented with the same questionnaire structure. | Methods – Survey Instrument and Domains |
| Survey administration | Number of items | The questionnaire contained 35 items. | Methods – Survey Instrument and Domains |
| Survey administration | Number of screens (pages) | The questionnaire was administered electronically through Microsoft Forms. The exact internal page or screen structure of the platform was not analysed separately for reporting purposes; participants progressed through the standard survey interface until final submission. | Methods – Survey Instrument and Domains |
| Survey administration | Completeness check | The questionnaire required completion of all mandatory items before submission. Selected open-ended items were optional and could be left blank or answered without additional comment. Platform-based checks prevented submission until mandatory items were completed. | Methods – Survey Instrument and Domains / Instrument Development and Theoretical Foundation |
| Survey administration | Review step | Participants were able to review and modify their responses before final submission using the standard Microsoft Forms interface. | Methods – Survey Instrument and Domains |
| Response rates | Unique site visitor | Unique site visitors were not tracked because the survey was distributed through WhatsApp rather than a publicly hosted website with visitor analytics. | Not applicable / not measurable in this survey design |
| Response rates | View rate | A view rate could not be calculated because unique visitors to the first survey page and unique visitors to the host communication platform were not tracked. | Not applicable / not measurable in this survey design |
| Response rates | Participation rate | The participation rate was estimated using the number of completed questionnaires divided by the approximate eligible trainee population in the London School of Paediatrics: 30/450 (6.7%). Because first-page access analytics were unavailable, a standard CHERRIES recruitment rate could not be calculated. | Results – Overview; Participant Characteristics |
| Response rates | Completion rate | Only fully submitted questionnaires were included in the dataset. Because separate first-page access and consent-page analytics were unavailable and the survey was not analysed as a multipage attrition design, a formal CHERRIES completion rate could not be calculated. | Methods – Survey Instrument and Domains; Results – Overview |
| Preventing multiple entries | Cookies used | Cookies were not used to assign unique identifiers or prevent duplicate entries. | CHERRIES appendix |
| Preventing multiple entries | IP check | IP addresses were not collected or used to identify duplicate entries, in order to preserve anonymity. | Methods – Ethical Considerations / CHERRIES appendix |
| Preventing multiple entries | Log file analysis | No log file analysis was undertaken to identify duplicate entries. | CHERRIES appendix |
| Preventing multiple entries | Registration | No username-based registration or login system was used. Access was limited pragmatically by distribution within the closed trainee groups. | Methods – Setting, Participants, and Recruitment |
| Analysis | Handling of incomplete questionnaires | Only fully submitted questionnaires were included in the analysis. Missing data within optional items, particularly open-ended responses, were treated as item-level non-response. | Methods – Data Analysis |
| Analysis | Questionnaires submitted with an atypical timestamp | Response times were not used as an exclusion criterion, and no cut-off based on questionnaire completion time was applied. | Methods – Data Analysis / CHERRIES appendix |
| Analysis | Statistical correction | No weighting, propensity score adjustment, or other statistical correction for non-representativeness was applied. Analyses were descriptive and exploratory, and findings were interpreted as hypothesis-generating rather than generalizable. | Methods – Data Analysis; Results – Overview; Strengths and Limitations |
